# Small intestinal submucosa-derived extracellular matrix as a heterotopic scaffold for cardiovascular applications

**DOI:** 10.3389/fbioe.2022.1042434

**Published:** 2022-12-12

**Authors:** Tiziana Palmosi, Anna Maria Tolomeo, Carmine Cirillo, Debora Sandrin, Manuela Sciro, Susanna Negrisolo, Martina Todesco, Federico Caicci, Michele Santoro, Eleonora Dal Lago, Massimo Marchesan, Michele Modesti, Andrea Bagno, Filippo Romanato, Paolo Grumati, Assunta Fabozzo, Gino Gerosa

**Affiliations:** ^1^ Laboratory of Cardiovascular Medicine, Department of Cardiac, Thoracic, Vascular Sciences and Public Health, University of Padova, Padua, Italy; ^2^ L.i.f.e.L.a.b. Program, Consorzio per la Ricerca Sanitaria (CORIS), Veneto Region Padua, Italy; ^3^ Telethon Institute of Genetics and Medicine (TIGEM), Pozzuoli, Italy; ^4^ Optics and Bioimaging Lab, Department of Physics and Astronomy, Fondazione Istituto di Ricerca Pediatrica Città della Speranza, University of Padova, Padua, Italy; ^5^ Azienda Ospedale Padova, Padua, Italy; ^6^ Laboratory of Immunopathology and Molecular Biology of the Kidney, Department of Women’s and Children’s Health, University of Padova, Padua, Italy; ^7^ Department of Industrial Engineering, University of Padova, Padua, Italy; ^8^ Department of Biology, University of Padova, Padua, Italy; ^9^ Consultant of Animal and Food Welfare, Padua, Italy; ^10^ Department of Physics and Astronomy “G. Galilei”, University of Padova, Padua, Italy; ^11^ Department of Clinical Medicine and Surgery, University of Napoli Federico II, Naples, Italy; ^12^ Cardiac Surgery Unit, Hospital University of Padova, Padua, Italy

**Keywords:** cardiac repair, decellularization, cardiac surgery, ECM, extracellular matrix, SIS, Triton (X-100), Tergitol 15-S-9

## Abstract

Structural cardiac lesions are often surgically repaired using prosthetic patches, which can be biological or synthetic. In the current clinical scenario, biological patches derived from the decellularization of a xenogeneic scaffold are gaining more interest as they maintain the natural architecture of the extracellular matrix (ECM) after the removal of the native cells and remnants. Once implanted in the host, these patches can induce tissue regeneration and repair, encouraging angiogenesis, migration, proliferation, and host cell differentiation. Lastly, decellularized xenogeneic patches undergo cell repopulation, thus reducing host immuno-mediated response against the graft and preventing device failure. Porcine small intestinal submucosa (pSIS) showed such properties in alternative clinical scenarios. Specifically, the US FDA approved its use in humans for urogenital procedures such as hernia repair, cystoplasties, ureteral reconstructions, stress incontinence, Peyronie’s disease, penile chordee, and even urethral reconstruction for hypospadias and strictures. In addition, it has also been successfully used for skeletal muscle tissue reconstruction in young patients. However, for cardiovascular applications, the results are controversial. In this study, we aimed to validate our decellularization protocol for SIS, which is based on the use of Tergitol 15 S 9, by comparing it to our previous and efficient method (Triton X 100), which is not more available in the market. For both treatments, we evaluated the preservation of the ECM ultrastructure, biomechanical features, biocompatibility, and final bioinductive capabilities. The overall analysis shows that the SIS tissue is macroscopically distinguishable into two regions, one smooth and one wrinkle, equivalent to the ultrastructure and biochemical and proteomic profile. Furthermore, Tergitol 15 S 9 treatment does not modify tissue biomechanics, resulting in comparable to the native one and confirming the superior preservation of the collagen fibers. In summary, the present study showed that the SIS decellularized with Tergitol 15 S 9 guarantees higher performances, compared to the Triton X 100 method, in all the explored fields and for both SIS regions: smooth and wrinkle.

## 1 Introduction

Cardiac structural diseases, either congenital or acquired, often require the use of prosthetic material for an appropriate reconstruction (Holubec et al., 2015). These patches of tissue may be synthetic or biological in nature, based on clinical application. For instance, Dacron® (Koch Industries, Inc., Wichita, KS, United States) (Vaideeswar et al., 2011) and the expanded polytetrafluoroethylene (ePTFE; WL Gore & Associates, Inc., Newark, DE, United States) (Terakawa et al., 2022), for their mechanical properties, water resistance, and suboptimal level of biocompatibility, are ideal to replace entire portions of great vessels and are adaptable to open surgery and endovascular, minimally invasive, delivery approaches (Hollister, 2005). Nevertheless, they cannot work as a regenerative scaffold and are exposed to a higher risk of complications compared to their biological counterpart, including mechanical failure, infection, and thrombosis (Vaideeswar et al., 2011). On the other hand, biological patches, such as autologous pericardium and xenografts, present greater compatibility and well-preserved three-dimensional architecture, namely, the extracellular matrix (ECM), which itself could favor implant internalization in the surrounding tissue (Li et al., 2011), improving patients’ quality of life and clinical outcomes as they are at lower risk of infection and do not require lifelong pharmacological therapy (e.g., aspirin). Unfortunately, the presence of cells and their remnants, although chemically pre-treated (e.g., glutaraldehyde), is demonstrated to be involved in the host’s inflammatory response, resulting in fibrosis and calcification that may lead to prosthetic dysfunction in the long term (Eybl et al. 1989; Huang-Lee et al. 1990). To overcome such limitations, in the last two decades, bioengineering strategies have been adopted and continuously implemented. They aim to effectively remove cells and their remnants from the biologically derived scaffold to reduce immune-induced processes of degeneration and facilitate host cells’ seeding to obtain functionalized and safely implantable supports (Carnagey et al., 2003). The so-derived ECM preserves its pleiotropic properties, presenting not only an intact three-dimensional structure, for an effective implant, but also favoring the development of functional tissue, at the injured site. This process, called “constructive remodeling”, defines the growth of native tissue adjacent to the implant and the restoration of the original function (Badylak, 2004). From a clinical standpoint, a versatile and functional scaffold, capable of remodeling induced by the site of the implant, is crucial to repair defects, especially in the pediatric population. Indeed, in these patients, reconstructive surgery is performed repeatedly since multiple surgical procedures are necessary during their life to adapt to the size of afunctional supports and treat devices’ failure (Weis et al., 2022). Therefore, an ideal prosthetic patch, independent of its application, should not only guarantee adequate physical and mechanical properties (strength, permeability, biocompatibility, etc.), also serve as a platform for further tissue tunable functionalization. SIS has been gaining great interest in this field. It is obtained from the jejunal portion of the pig’s small intestine and due to its great versatility, has proven to have promising characteristics as a constructive remodeling scaffold (Hodde et al., 1996; Chen and Badylak, 2001; Lindberg and Badylak, 2001; Padalino et al., 2012; Li et al., 2018). In particular, SIS is known to be rich in growth factors such as vascular endothelial cell growth factor (VEGF) (Hodde et al., 2001), basic fibroblast growth factor (b-FGF) and transforming growth factor beta (TGF-β) (Mcdevitt et al., 2003). It also contains 90% collagen (predominantly type I and IV), fibronectin, laminin, and glycosaminoglycans (Mcpherson and Badylak, 1998; Badylak, 2004). CorMatrix® is an acellular SIS-ECM, initially marketed for pericardial closure; its application was approved by the FDA in 2010 for the repair of intracardiac, aortic, and great vessel defects due to its high maneuverability. To date, CorMatrix® is used for cardiac and extra-cardiac applications.

Although several studies have reported encouraging results (Badylak et al., 1989; Lantz et al., 1992; Fallon et al., 2012; Padalino et al., 2012; Baker et al., 2019), incomplete cellularity of the final product has been observed, which is responsible for immune rejection, calcification, and fibrosis (Woo et al., 2016; Nelson et al., 2016; Sood et al., 2021). Therefore, the clinical results obtained with the use of CorMatrix are controversial (Quarti et al., 2011; Rosario-Quinones et al., 2015; Nezhad et al., 2017; Sengupta et al., 2022; Ma, 2022). A more sophisticated and specific type of decellularization process, associated with a dedicated sterilization protocol, will contribute to optimizing SIS performance. Our group has previously developed a protocol for decellularization based on Triton-X 100 (X-100) detergent, and its efficacy has been confirmed on several cardiac tissues (Spina et al., 2003; Bottio et al., 2008; Naso et al., 2010; Gallo et al., 2012; Aguiari et al., 2017; Iop et al., 2017; Zouhair et al., 2020). Triton-X 100 has been removed from the market since January 2021 for its endocrine-disrupting properties (ECHA, REACH), and a new protocol has been developed by our group, which is based on a new detergent called Tergitol 15-S 9. This is a non-ionic, linear-chain surfactant belonging to the series of ethoxylated secondary alcohols, which has received promising preliminary results in the decellularization process (Faggioli et al., 2022; Todesco et al., 2022; Casarin et al., 2022). Unlike Triton X-100, Tergitol 15-S 9 is easily biodegradable, does not induce cytotoxicity, and, due to its chemical structure, has a much faster micelle formation mechanism, resulting in better wettability.

With this in mind, the aims of this work are as follows: 1) to describe the histological, biochemical, and biomechanical properties of the two portions (wrinkled and smooth) isolated from the SIS before and after the decellularization processes; 2) to compare the two decellularization protocols and finally validate the Tergitol-based one; and 3) to demonstrate, through a fine proteomic characterization, that the decellularized SIS can keep not only a solid ultrastructure but also adequate functional properties, promising for its repopulation *in vivo*.

## 2 Materials and methods

### 2.1 Tissue collection: Smooth and wrinkled SIS isolation

Native pig intestines were collected from a local Duroc pig slaughterhouse, weighing between 140 and 170 kg and aged between 9 and 14 months. The protocols followed by the slaughterhouse were consistent with EC regulations 1099/2009 regarding animal health and protection, at the time of slaughter, supervised by the Italian government and approved by the associated legal authorities of animal welfare (Food and Consumer Product Safety Authority).

A proximal portion of the jejunum was taken from each animal (*n* = 6). The porcine intestinal submucosa was isolated by mechanical detachment of the serosa, the membrane that covers it externally (Badylak et al., 1989). At this point, both smooth and wrinkled portions were visible. Subsequently, the submucosa was cut longitudinally between the two smooth and wrinkled portions, and the two decellularization processes were performed.

### 2.2 Decellularization protocol with Triton X 100 and Tergitol 15 S 9 and thickness

SIS tissue samples were decellularized with Triton X 100 and Tergitol 15 S 9 protocol and finally were cut in circular punches (0.8 cm of diameter, Kai Medical) in two different directions: longitudinally and circumferentially.

To assess whether decellularization can influence the geometrical characteristics of the pig tissue, the thicknesses of smooth and wrinkled scaffolds were measured using a digital caliper (model ID-C112XB, Mitutoyo America Co, Aurora IL, United States) before and after both decellularizations.

The decellularizations of the swine SIS samples (*n* = 6 smooth and *n* = 6 wrinkled for each treatment) were performed in parallel. The first decellularization protocol, i.e., the TriCOL method, was based on the combined action of the non-ionic detergent Triton X 100 (X 100, Merk) and sodium cholate (C1254, Merk); this method has been developed by our group (Spina et al., 2003). The second decellularization protocol used the same procedure, but we replaced Triton X 100, which, in December 2012, was added to the candidate list for authorization by the European Chemical Agency (ECHA) as a substance with an equivalent level of concern due to its degradation into a byproduct with endocrine-disrupting properties, with another non-ionic detergent, i.e., Tergitol 15 S 9 (15 S 9, Merk). Briefly, in both decellularization protocols, all porcine samples (smooth and wrinkled) were treated using protease inhibitors, alternating hypo- and hypertonic solutions with increasing concentrations (0.1%–1% w/v) of Triton X 100 and Tergitol 15 S 9 and 10-mM sodium cholate (C1254, Merk). All solutions were mixed with a pH-buffering solution containing 10 mM sodium ascorbate (A7631, Merk) and 5 mM ethylenediaminetetraacetic acid (EDTA) (E9884, and Merk) under continuous agitation. Residual nucleic acids were digested using 1500 U/cm^2^ of Benzonase™ nuclease, a non-specific endonuclease (E1014, Merk) at 37°C for 48 h.

### 2.3 High-level disinfection

This method was developed by our group for bovine and porcine pericardia, as previously reported (Fidalgo et al., 2018), and adapted in the present work for the intestinal tissue (Supplementary Figure S1A). To reduce the bioburden physiologically present in the intestinal tract, the samples were treated with a combination of antibiotics, vancomycin hydrochloride (V0045000, European Pharmacopoeia-EP, Merk), cefoxitin sodium (C0688000, European Pharmacopoeia-EP, Merk), and gentamicin sulphate (G1397, European Pharmacopoeia-EP, Merk). In addition, to increase enterobacterial decontamination power, another antibiotic, i.e., ciprofloxacin (17850, Merk) (Albuquerque et al., 2015), was included. Finally, the antimycotic, i.e., amphotericin B (Y00005, European Pharmacopoeia-EP Merk), was added to the cocktail. The pSIS samples (smooth and wrinkled) were subjected to AA treatment at a temperature of 37 C for 24 h in agitation in a plate shaker. They were then washed with sterile PBS to remove antibiotic and antimycotic residues from the tissue. Subsequently, they were tested for sterility, as described in the following section.

### 2.4 Sterility assessment

Sterility tests were performed in accordance with the guidelines of European Pharmacopoeia 2.6.1 for biological specimens (EP 2.6 sterility test; EP 2.6 Biological tests, 01/2005:20601; European Pharmacopoeia, 2005) by dipping the SIS scaffolds in two bacterial culture mediums: thioglycolate medium (THIO, Biolife) and tryptone soya broth (TSB, Biolife) (Supplementary Figure S1).

The THIO medium is mainly used for the culture of anaerobic bacteria; however, it also detects aerobic bacteria, while TSB is suitable for the culture of fungi and aerobic bacteria.

The culture media were prepared according to the manufacturer’s protocol, autoclaved at 121°C for 2 h, and stored at a temperature of +4°C until use.

At the end of each decellularization and disinfection treatment, samples from each animal were immersed in both media. Specifically, they were incubated for up to 14 days at 35°C for the THIO medium and at 22°C for the TSB medium. Only medium was used as a control.

At the end of the incubation period, the turbidity of the medium was examined by visual inspection (EP 2.6 sterility test) by assuming turbid media indicative of contamination and clear, sterile media (Supplementary Figure S2).

### 2.5 DNA quantification

DNA quantification was performed on SIS porcine samples (*n* = 6 smooth and *n* = 6 wrinkled) with a dry weight ranging between 15–and 20 mg. DNA extraction was performed with the DNeasy® Blood & Tissue kit (69504, Qiagen) after the lyophilization process. Subsequently, the concentration was measured with a NanoDrop 2000 spectrophotometer (ThermoFisher Scientific) at a wavelength of 260 nm. The final DNA amounts were expressed in ng dsDNA/mg dry tissue.

### 2.6 Histological analysis

Histological evaluations were assessed by staining smooth and wrinkled SIS samples, both native and decellularized (*n* = 6 smooth and *n* = 6 wrinkled), with Triton X 100 and Tergitol 15 S 9. All staining was performed on paraffin-embedded samples (Histo-Line laboratories), working with a temperature of 58°C, after dehydration with Citro Histoclear (Histo-line laboratories). Finally, we cut the tissues with a manual rotary microtome (Histo-Line laboratories) to a thickness of 6–7 μm using a water bath desiccator to keep the paraffin-embedded sample slices intact at a temperature of 45°C. Hematoxylin and eosin (H&E; 04-061010, Bio-Optica), Picrosirius red (PR), Masson’s trichrome (MT; 04-010802, Bio-Optica), and Weigert-Van Gieson (WvG; 04-052812, Bio-Optica) were performed following the detailed procedure reported in the Technical Bulletins. Images were acquired with an optical microscope (Olympus BX51, Olympus Corporation, Tokyo, Japan) equipped with a Nikon Eclipse 50i camera and NIS-Elements D 3.2 software (Nikon Corporation Shinagawa).

### 2.7 Immunofluorescence alpha-gal staining

Native and decellularized samples of porcine SIS, smooth and wrinkled (*n* = 6 for each) (Supplementary Figure S3), were cut with a circular puncher (0.8 cm diameter, Kai Medical) and embedded in the following: 1) 1:1 solution of O.C.T. (optimal cutting temperature compound, 4583, Sakura Tissue- Tek); 2) 20% sucrose (S0389, Merk) for 24 h; 3) frozen in liquid nitrogen; and 4) isopentane fumes (2-methylbutane, 277258, Merk) and stored at −80°C. The cryosections were cut to a thickness of 7 μm on a cryostat (Leica Biosystems) and indirect immunofluorescence was performed to assess the presence status of the alpha-gal epitope in the matrix, before and after decellularization treatments with the two different detergents. The primary antibody LSBio (LS-C634159 clone M86 1:10) was used. The secondary antibody was Alexa Fluor 594 anti-mouse IgG (red, 1:200, Millipore). Images were acquired with confocal ZEISS LSM 800 microscope Zeiss, Germany).

### 2.8 Biochemical profile

#### 2.8.1 Hydroxyproline

As hydroxyproline is one of the most abundant amino acids in collagen, its detection can be used as an indicator of collagen content in freeze-dried samples of native and decellularized SIS (*n* = 3 for each). In the hydroxyproline test kit (MAK008, Merk), the concentration of hydroxyproline is determined by the reaction of the oxidized amino acid with 4-(dimethylamino) benzaldehyde (DMAB), which results in a colorimetric product (560 nm), whose concentration is directly proportional to the hydroxyproline content. The absorbance was measured immediately with an absorbance microplate reader (Sunrise™ Tekan).

#### 2.8.2 Sulphated glycosaminoglycans

Sulphated glycosaminoglycans (i.e., heparan sulphate, dermatan sulphate, and chondroitin sulphate), which are essential for driving growth factors in tissue remodeling, were quantified using the Blyscan Glycosaminoglycan Sulphate Assay Kit (B1000, Biocolor). Samples of native and decellularized SIS (*n* = 3 smooth and *n* = 3 wrinkled) were lyophilized and digested with papain extraction solution (P3375, Merk) at 60°C. Aliquots of each digested sample were mixed with 1,9-dimethyl-methylene blue dye (DMMB) to precipitate the sGAGs-dye complex. A dissociation reagent was then used to release the bound dye into the solution, and its absorbance was measured at 656 nm in a microplate reader (Sunrise™ Tekan).

#### 2.8.3 Elastin

Elastin, a component of the connective tissue, enables body tissues to return to their original shape after being subjected to stretching or contracting forces. It was quantified using the Fastin Elastin assay kit (F2000, Biocolor). Samples of porcine SIS (*n* = 3 smooth and n = 3 wrinkled) were freeze-dried and treated two times, with 0.25 M oxalic acid at 100°C for 1-h each, to extract α-elastin. The extracts were combined with Fastin reagent (5,10,15, 20- tetraphenyl-21H, 23H-porphyrin tetrasulphonate (TPPS). Guanidine HCl, dye dissociation reagent and propan-1-ol were then added to release the dye into the solution, the absorbance of which was measured at 513 nm in a microplate reader (Sunrise™ Tekan).

### 2.9 Differential scanning calorimetry

The shrinkage temperature was determined by differential scanning calorimetry (DSC) analysis. Thermograms were recorded with a DSC Q200 calorimeter (TA Instruments, New Castel, Delaware, United States). Samples (10–15 mg) were dried and sealed into aluminum pans. Each sample was brought to 20°C, and scans were performed from 20 to 90°C with a heating rate of 10°C min^−1^ (Zouhair et al., 2020). Empty pans were used as references.

### 2.10 Two-photon microscopy

Second harmonic generation (SHG) imaging was performed on smooth and wrinkled SIS samples, both native and decellularized (*n* = 6 each), using a custom-developed multiphoton microscope previously described by Filippi et al. (2018). Briefly, an incident wavelength of 800 nm was adopted to detect the SHG signal of collagen at 400 nm on the photodetector (GaAsP PMT with a 395/25 nm bandpass filter). Z-stack images were acquired, at a fixed magnification, through the Olympus 25X water-immersion objective with a numerical aperture of 1.05 (1,024 × 1,024 pixels), with an average of 80 consecutive frames, a pixel dwell time of 0.14 μs, and a pixel width of 0.8 μm. By combining two-photon excited autofluorescence and SHG, quantitative measurements were obtained. The coherence of collagen fibers was calculated to verify the dominant local orientation of the images using OrientationJ (http://bigwww.epfl.ch/demo/orientation/), a plugin of ImageJ, as described by Rezakhaniha et al. (2012). The estimated parameter was bounded between 0 and 1, indicating the absence (isotropy) and presence (anisotropy) of a dominant orientation, respectively. Fast Fourier transform (FFT) was used to describe the structure of the tissue matrix. It converts the SHG image into a new shape using the spatial frequency properties of pixel intensity variations, allowing textural features to be extracted from the image and showing fiber organization and distribution. Indeed, a fiber strongly oriented in a single direction shows an elliptical shape; conversely, a circular shape represents a fiber spread in all directions (Borile, 2021; Wu et al., 2011). For quantitative measurements, the uncompressed RAW images were analyzed with Image-J software (Shindelin, 2012).

### 2.11 Scanning electron microscopy

To characterize the influence of the two different detergents on porcine SIS, the morphology of the tissue surface was evaluated by SEM microscopy (JEOL JSM-6490, Peabody, MA, United States), with a particular focus on the dynamism of collagen, which is the main component of the dECM of SIS. Circular patches, from native and decellularized samples, both smooth and wrinkled (*n* = 6 for each), were previously fixed in Karnovsky solution (8% (w/v) PFA, 10% (v/v) GA (50%), and 40% (v/v) cacodylate buffer (0.2 M), pH range 7.2–7.4; all reagents were supplied by Merk) at +4°C in the dark. Prior to analysis, tissues were rinsed in PBS (phosphate buffer saline; 137 mM sodium chloride, 2.7 mM potassium chloride, 10 mM disodium phosphate, 10 mM potassium phosphate pH 7.4, all supplied by Merk) and dehydrated with ascending ethanol solutions (70%, 80%, 90%, and 100%) for 10 min each. They were then dried under vacuum in a critical point dryer (Polaron CDP7501) to replace ethanol with liquid CO_2_ and avoid tissue damage. Finally, SIS samples were metalized with gold to create a conductive layer on the surface of the samples with Edwards S150B sputter coater. Images were acquired in low vacuum mode at 20 kV, at different magnifications.

### 2.12 Transmission electron microscopy

Small pieces of tissue samples (about 2–3 mm^3^), (*n* = 6 smooth and *n* = 6 wrinkled) were fixed with 2.5% glutaraldehyde plus 2% paraformaldehyde in 0.1 M sodium cacodylate buffer pH 7.4 overnight at 4°C. Subsequently, the samples were postfixed with 1% in 0.1 M sodium cacodylate buffer for 1 h at 4°C. After three water washings, the samples were dehydrated in a graded ethanol series and embedded in epoxy resin (Merk). Ultrathin sections (60–70 nm) were obtained with a Leica Ultracut EM UC7 ultramicrotome, counterstained with uranyl acetate and lead citrate, and viewed using a Tecnai G (FEI) transmission electron microscope operating at 100 kV. Images were captured using a Veleta (Olympus Soft Imaging System) digital camera.

### 2.13 Biomechanical analysis: Uniaxial tensile test

The biomechanical analysis was performed using the TRAMA (TRAction MAchine) system (IRS, Ingegneria Ricerca Sistemi, Padua), whose movement and data acquisition are operated by a dedicated Labview software (National Instrument, United States). The system has four linear actuators, each equipped with a load cell (50 N). Native and decellularized samples were biomechanically assessed. For each tissue, smooth and wrinkled regions were analyzed (*n* = 3 each). Each sample was cut in dog bone shape with a gauge length of 5 and 2 mm width by means of an in-house designed cutter.

Samples were tested in two directions: longitudinal and circumferential, parallel to the main axis of the intestinal lumen and perpendicular to it, respectively.

Sample thickness was measured using a Mitutoyo digital caliber (model ID-C112XB, Mitutoyo America Co, Aurora, IL, United States) by sandwiching them between two glass slides, whose thickness was then subtracted.

Samples were thus preloaded up to 0.1 N and then extended with an elongation rate of 0.2 mm/s (0.1 mm/s per actuator) until failure. Tests were performed at room temperature, and samples were continuously wetted with 0.9% w/v NaCl in order to avoid dehydration.

Data were analyzed by an in-house developed Matlab® script (Mathworks, Natick, MA, United States) to measure ultimate tensile strength (UTS) as the maximum resistance to failure, failure strain (FS) as the maximum elongation of the samples, and Young’s modulus (E) as the slope of stress and strain curve in the linear region(s). Stress was obtained by dividing the load by the original area of the cross-section of the specimen, while strain was calculated by dividing the elongation of the gauge length of the specimen by its original area (Todesco et al., 2021).

### 2.14 *In vitro* viability and cytotoxicity assays

Static cell seeding on 96-well polystyrene plates for tissue culture (Costar®, Corning Incorporated) and dimethyl sulfoxide, DMSO (276855, Merk), was performed with human umbilical vein endothelial cells (HUVECs) at passage 5 and cultured under standard conditions. For endothelial cells, a culture medium containing supplemented and growth medium (EGM™-2 Endothelial Cell Growth Medium-2 BulletKit™, CC3162, and Lonza) was used. All samples and controls were seeded in triplicate with HUVECs at a density of 1,00,000 cells/cm^2^. Briefly, smooth and wrinkled decellularized SIS dECM samples were seeded with cells. Positive cytotoxicity controls consisted of cells seeded alone, without any SIS dECM samples, while negative controls consisted of cells seeded onto smooth and wrinkled tissue treated with SIS dECM supplemented with DMSO. The timepoints were 1, 3, and 7 days.

### 2.15 Cell viability/mortality qualitative test

A live/dead staining kit (MP03224, kit for mammalian cells, Molecular Probes) was used on decellularized scaffolds with both protocols, in smooth and wrinkled tissues, to evaluate the behavior of the endothelial cells in different conditions. The test was performed according to the manufacturer’s fluorescence microscopy protocol. At the end of each timepoint, the results were analyzed by using a Leika DM4500B microscope coupled with a DFC320R2 camera for image acquisition.

### 2.16 Angiogenesis assay

To assess the angiogenesis capacity of endothelial cells *in-vitro*, all decellularized samples (obtained with decellularization protocols, Triton X 100 e Tergitol 15 S 9, in smooth and wrinkled tissue) and controls were seeded in triplicate with human umbilical vein endothelial cells (HUVECs) on Matrigel™ Matrix (Corning) at a density of 13,000 cells/cm^2^, using 96-well plates with treated tissue culture surfaces (Costar®, Corning Incorporated). All steps were performed under sterile conditions. Briefly, the SIS-dECM tissue samples, after treatments, smooth and wrinkled, were homogenized with an electric rotary homogenizer at 35,000 rpm (Cole-Parmer LabGEN 7) by adding the complete medium with supplements and growth factors (1 mg treated tissue/1 ml medium) and stored for 24 h at 37°C, 5% CO_2_. Subsequently, HUVEC cells were seeded on Matrigel^TM^ and plated with the conditioned medium with homogenized SIS dECM. In positive control, cells were seeded on Matrigel ^TM^ and plated with complete endothelial cell culture medium. A non-specific cell culture medium, DMEM (Fisher Scientific), was used as a negative control. Tubule formation occurred within 4 h, at 37°C, 5% CO_2_, and was analyzed under the EVOS TM XL CORE PROMO microscope (MM Biotech). The extent of the newly formed tubules was quantified by measuring the total length of the network formed between the cells using ImageJ software (Angiogenesis macro).

### 2.17 Mass spectrometry

All experiments have been performed in a labeling-free setting. Biopsies from tissues were lysate in RIPA buffer implemented with protease inhibitors. Thirty micrograms of total protein lysate were precipitated with cold acetone and then reduced and alkylated in a solution of 6 M Guanidine-HCl, 5 mM TCEP, and 55 mM chloroacetamide. Peptides were obtained by digesting proteins with LysC (WAKO) for 3 h at 37°C and with the endopeptidase sequencing-grade trypsin (Promega) overnight at 37°C. Collected peptide mixtures were concentrated and desalted using the Stop and Go Extraction (STAGE) technique (Rappsilber et al., 2003a; Rappsilber et al., 2003b).

Instruments for LC-MS/MS analysis consisted of a NanoLC 1200 coupled via a nano-electrospray ionization source to the quadrupole-based Q Exactive HF benchtop mass spectrometer (Michalski et al., 2011). Peptide separation was carried out according to their hydrophobicity on a home-made chromatographic column, 75 µm ID, 8 Um tip, 350 mm bed packed with Reprosil-PUR, C18-AQ, 1.9 µm particle size, 120 Å pore size, using a binary buffer system consisting of solution A: 0.1% formic acid and B: 80% acetonitrile and 0.1% formic acid.

Runs of 120 min after loading were used for proteome samples, with a constant flow rate of 300 nl/min. After sample loading, run start at 5% buffer B for 5 min, followed by a series of linear gradients, from 5% to 30% B in 90 min, then a 10 min step to reach 50% and a 5 min step to reach 95%. This last step was maintained for 10 min.

Q Exactive HF settings: MS spectra were acquired using 3E6 as an AGC target, a maximal injection time of 20 ms, and a 120,000 resolution at 200 m/z.

The mass spectrometer operated in a data-dependent top-20 mode with subsequent acquisition of higher-energy collisional dissociation (HCD) fragmentation MS/MS spectra of the top 20 most intense peaks. The resolution for MS/MS spectra was set to 15,000 at 200 m/z, AGC target to 1E5, max injection time to 20 ms and the isolation window to 1.6 Th. The intensity threshold was set at 2.0 E4 and dynamic exclusion at 30 s.

### 2.18 Data analysis

For mass spectrometry, all acquired raw files were processed using MaxQuant (Tyanova et al., 2016a) and the implemented Andromeda search engine. For protein assignment, spectra were correlated with the UniProt Sus scrofa database including a list of common contaminants. Searches were performed with tryptic specifications and default settings for mass tolerances for MS and MS/MS spectra. Carbamidomethyl at cysteine residues was set as a fixed modification, while oxidations at methionine and acetylation at the N-terminus were defined as variable modifications. The minimal peptide length was set to seven amino acids and the false discovery rate for proteins and peptide-spectrum matches to 1%. The match-between-run feature with a time window of 1 min was used. For further analysis, the Perseus software (1.6.2.3) (Tyanova et al., 2016b) was used and first filtered for contaminants and reverse entries and proteins that were only identified by a modified peptide. The LFQ intensities were logarithmized, and data visualization was carried out in the statistical environment R (Cox 2014).

The mass spectrometry proteomics data have been deposited to the ProteomeXchange Consortium (Deutsch et al., 2020) *via* the PRIDE (Perez-Riverol et al., 2022) partner repository with the dataset identifier PXD038206.

### 2.19 Statistical analysis

Continuous variables were expressed as mean ± SD. One-way ANOVA was conducted using Tukey and Bonferroni’s multiple comparisons test and was used for multiple comparisons. Data were analyzed using GraphPad 9 software. *p* values: *p < 0.05; **p < 0.01; ***p < 0.005; ****p < 0.0001.

## 3 Results

### 3.1 Effectiveness of decellularization protocols

**FIGURE 1:**
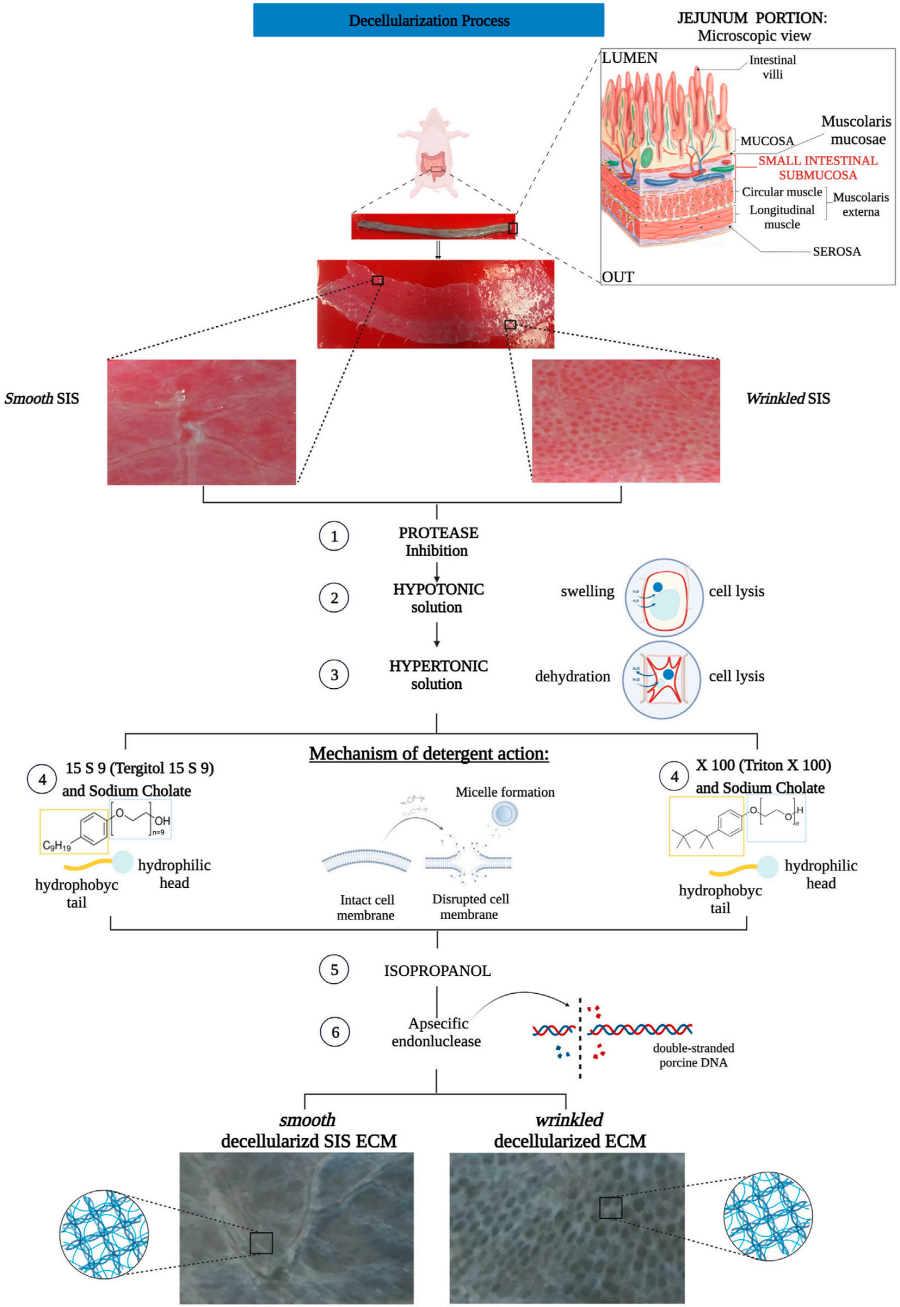
Flow chart decellularization process with Triton X 100 and Tergitol 15 S 9 of smooth and wrinkled SIS. The tubular tissue of SIS was extracted between the mucosa (internally) and the serosa (externally). The tube was opened and separated into its two different regions, smooth and wrinkled, and subjected to decellularization processes. Both involved a pre-treatment with a solution that inhibited the action of proteases to block the enzyme activity that degrades the ECM. Subsequently, the use of hypo- and hyper-tonic solutions, with different salt concentrations, ensured cell lysis by swelling and dehydration of the cell, respectively. At step 4 of the decellularization protocols, the SIS tissues were separated, *n* = 6 smooth and wrinkled with Triton X 100 and *n* = 6 smooth and wrinkled with Tergitol 15 S 9. Both decellularization treatments share the mechanism of action: the detergent, through the hydrophilic head and hydrophobic tail, inserts itself between the phospholipids of the cell membrane, forming micelles and causing the disruption of the plasma membrane. Subsequently, treatment with isopropanol and an endonuclease was essential to remove the remaining phospholipid residues and inactivate the nuclear residues from the tissue.

**FIGURE 2:**
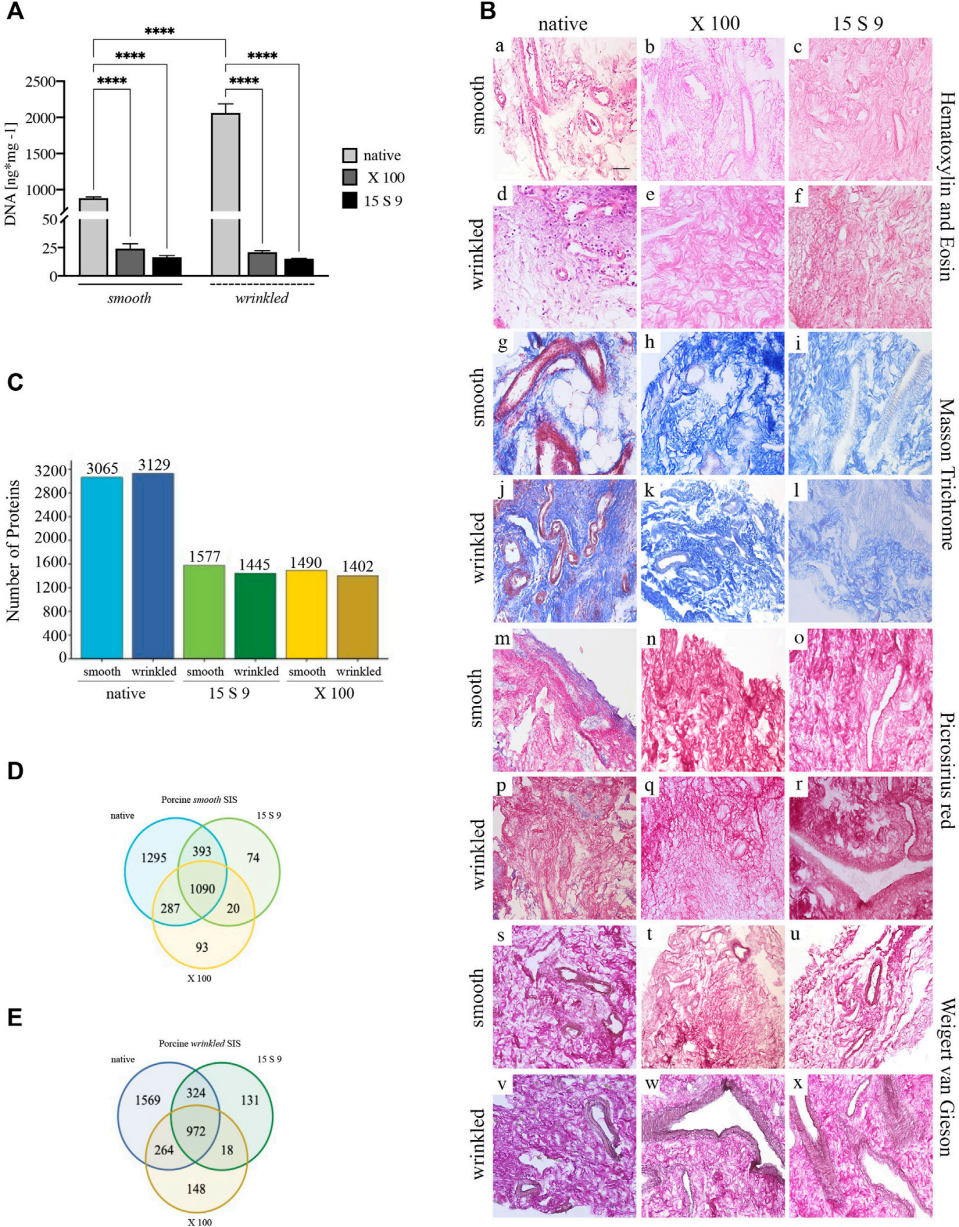
Yield and preservation of SIS tissue before and after decellularization with X 100 and 15 S 9. **(A)** DNA quantification. The amount of DNA (*n* = 6 for each) was significantly reduced, compared to the native, in the smooth and wrinkled portion after X 100 and 15 S 9 (*p-*value <0.0001), although the number of initial cells in the native wrinkled was significantly higher than in the native smooth (*p-*value <0.0001). **(B)**. Hematoxylin and eosin (a–f). A retained ECM was present after both decellularizations (in pink). In contrast to the native parts (smooth, a; wrinkled, d), the nuclei (in purple) were completely absent in the decellularized tissues (smooth b, c and wrinkled e, f: X 100 and 15 S 9, respectively). Masson’s trichrome (g–l). The organization and preservation of collagen fibers (in blue) together with the vascular apparatus (in red) were maintained after both decellularizations (smooth h, i and wrinkled k, l: X 100 and 15 S 9, respectively). Nuclei (in black brown) were absent in the decellularized, compared to the native (smooth, g; wrinkled, j). Picrosirius red (m–r). The morphology of the collagen network and vessels (in red) was preserved after decellularization (smooth n, o and wrinkled q, r: X 100 and 15 S 9, respectively) compared to the natives (smooth m; wrinkled p). Weigert-Van Gieson (s–x). No change in collagen fibers (in red) and elastic fibers around the vessels mainly (in black) of the decellularized ECM (smooth t, u and wrinkled w, x: X 100 and 15 S 9, respectively) compared to the native (smooth s; wrinkled, v). **(C)** Full Proteome analysis. A higher amount of protein content was present in the native wrinkled with respect to decellularized tissues. Venn diagrams of proteins regulated in **(D)** porcine smooth and **(E)** wrinkled compared to the native tissue. Scale bar: 100 um. Magnification: 20X

In smooth and wrinkled portions of the SIS tissue, DNA quantification confirmed the efficiency of decellularization after the use of both detergents (Figures 1, 2). The native wrinkled portion had more nucleic acid residues than its native smooth part (*p* value < 0.0001). After Triton X 100 (X 100) and Tergitol 15 S 9 (15 S 9) decellularizations, in the smooth and wrinkled tissues, the values of DNA were significantly lower (*p* value < 0.0001). In both portions, the quantity of nucleic acids was below the threshold value (50 ng ds DNA/mg dry tissue), thus fulfilling the decellularization efficiency criteria (Crapo et al., 2011). These data demonstrated equal effectiveness of both decellularization protocols in removing nucleic acids from treated tissues.

Histological analysis with H&E (Figure 2B, [a–f]) showed a densely vascularized ECM, similar in both smooth (in pink, [a]) and wrinkled (in pink, [d]) tissue portions, and a larger nuclear component was clearly detected in the latter (in purple, [d]). After decellularization, the nuclear component was not present in the tissue treated with X 100 (smooth, [b]; wrinkled, [e]) and with 15 S 9 (smooth, [c]; wrinkled, [f]). The ECM connective tissue (in pink) was preserved in both vascular tree and collagen bundles’ typical wavy structure, leaving the texture of the ECM, in its smooth and wrinkled portions, untouched after the use of both detergents (with X 100 [b–e]; 15 S 9 [c–f]).

Sterility tests, performed in accordance with the guidelines of European Pharmacopoeia 2.6.1 for biological specimens (EP 2.6 sterility test; EP 2.6 Biological tests, 01/2005:20601; European Pharmacopoeia, 2005), confirmed the sterility of both treated SIS-dECM (Supplementary Figures S1, S2).

To evaluate the potential immunogenicity of the treated SIS-dECM scaffolds, we analyzed the presence of alpha-gal by immunofluorescence assay. The alpha-gal epitope was present in large amounts in the native tissues, equally in the smooth and wrinkled parts, following a linear trend to be more expressed along the edges of the sample. DAPI-stained nuclei (blue) were present, apparently to a greater extent in the wrinkled than in the smooth tissue. After decellularization X 100, the epitope was still massively present compared with the native, and few nuclei (blue) were still present, mainly in the wrinkled. On the other hand, in the 15 S 9-treated samples, the fluorescence signal of alpha-gal decreased (Supplementary Figure S3).

### 3.2 ECM preservation of SIS tissues after decellularization

Smooth and wrinkled portions of the SIS were characterized to compare the effectiveness of the two detergents, namely, X 100 and 15 S 9, in decellularizing action and in preserving the ECM and its components. The two portions were compared before and after the use of X 100 and 15 S 9 and stained with Masson trichrome (Figure 2B, MT, smooth [g-i]; wrinkled [j-l]), Picrosirius red (Figure 2B, PR smooth [m-o]; wrinkled [p-r]), and Weigert-Van Gieson (Figure 2B, WvG smooth [s-u]; wrinkled [v-x]). From each staining, the native ECMs of the connective tissue in the two portions were similar. A dynamic collagen morphology was present throughout the smooth (with MT in blue [g] with PR and WvG in red [m and s]) and wrinkled (with MT in blue [j] with PR and WvG in red [p and v]) region, accompanied by rich vascularization in both (smooth: with MT, PR in red [g, m] and with WvG in brown, [s]) (wrinkled: with MT, PR in red [j, p] and with WvG in brown [v]). This scenario included the presence of arterioles and venules and, even after decellularization, the two portions retained similar characteristics. A conserved dense vascular network was still present after X 100, in both smooth (with MT in light blue [h], with PR and WvG in red [n, t]) and wrinkled portions (with MT in light blue [k], with PR and WvG in red [q and w]), but visibly better after 15 S 9 (smooth: with light blue MT [i], with PR and WvG in red [o and u]) (wrinkled: with light blue MT [i], with PR and WvG in red [r and x]), indicating a more conservative deterrent action. The nuclear component, present in both native tissues between the collagen fibers and around the vessels, was absent after decellularization treatment. The typical wavy morphology of the collagen fibers, in the connective tissue, was preserved after each treatment: no breaks or holes in the ECM.

### 3.3 Preservation of protein content after decellularization treatments

A similar protein content was detected by mass spectrometry (LC MS/MS) in the native wrinkled compared to smooth portion (Figure 2C). After treatments, there was a significant reduction of protein detection in treated samples compared to the native ones. 15 S 9 and X 100 treated samples showed similar number of remaining proteins, with a slight increase in protein number in 15 S 9 treated samples (Figure 2C).

Venn diagrams highlight the number of proteins that were conserved or not before and after as well as between the two different treatments (Figures 2D,E). After decellularization, the retained number of proteins was higher in the wrinkled compartment than in the smooth one (after X 100 = 148 vs. 93 and after 15 S 9 = 131 vs. 74). After 15 S 9 decellularization, the number of common proteins was higher in the smooth compartment than in the wrinkled one (1090 vs. 972). These results showed higher retention of common proteins between native and smooth X 100 decellularized tissue compared to the wrinkled one (287 vs. 264). A similar result was obtained after the 15 S 9 treatment in the smooth rather than in the wrinkled compartment (393 vs. 324).

### 3.4 Study of ultrastructure: Focus on collagen

Label-free two-photon microscopy is a useful type of imaging to evaluate scaffold structures without treating the tissue, thus avoiding damage. With this technique, the second harmonic generation (SHG) signal can be identified. The SHG value provides information not only on the preservation of the structural protein integrity of the ECM but also on the organization of the fibrillar structures of SHG-active molecules, in particular, collagen.

**FIGURE 3:**
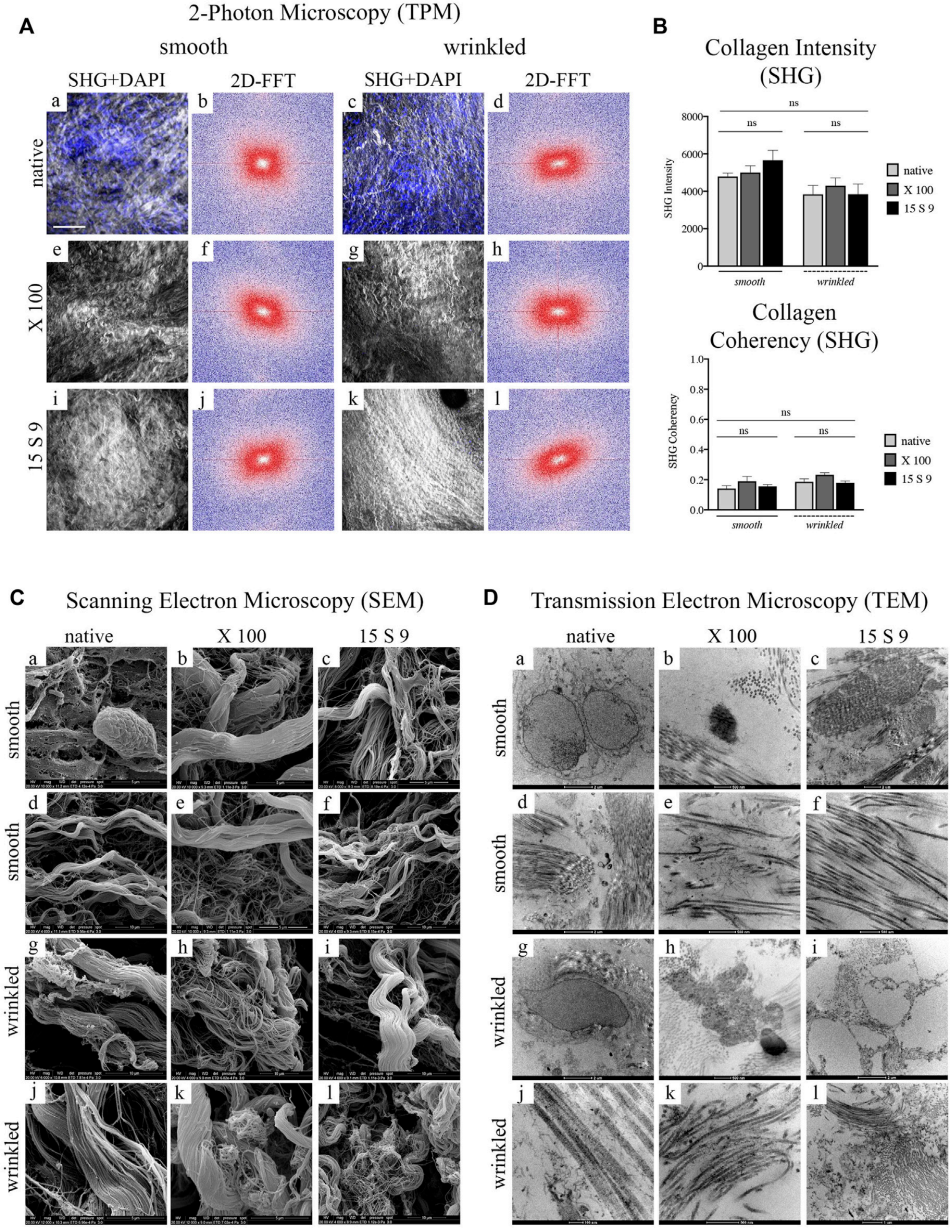
Ultrastructure analysis of collagen fibers after decellularization with X 100 and 15 S 9 in smooth and wrinkled tissue **(A)** SHG signal and 2D-FFTdiagrams. The SHG signal (*n* = 6 for each), white and grey staining in the images, of the native tissue (smooth, a; wrinkled, c) was characterized by an apparently disorganized fibrillar morphology, confirmed by the 2D-FFT images, (smooth, b; wrinkled, d). The 2D-FFT analysis of the smooth and wrinkled native showed a circular shape representation: this is the result of the absence of a single directionality of the native collagen fiber bundles. The nuclei, stained in blue by the DAPI probe, in the native tissues (smooth, a; wrinkled, c) followed the apparently disordered course of the collagen fibers. After decellularization with X 100 (smooth, e; wrinkled, g) and with 15 S 9 (smooth, i; wrinkled, k), the SHG and DAPI signal presented a well-preserved ECM without fiber damage and disruption. A greater alignment of the fibrillar pattern was evident from 2D-FFT, after treatments with X 100 (smooth, f; wrinkled, h) and 15 S 9 (smooth, j; wrinkled, l), compared to the native. Thus, the representations of the two-dimensional FFT diagrams had an elliptical shape, characterized by the identification of a main directionality of the fibers. Scale bar = 100 m. SHG: second harmonic generation. DAPI: 4′,6-diamidine-2-phenylindole. 2D-FFT: two-dimensional Fourier transform. X 100: Triton X 100.15 S 9: Tergitol 15 S 9. The SHG images, in the smooth and wrinkled portions, native and after decellularization, represent the most significant regions of interest (ROI) selected during the analysis to derive the 2D FFT plot (*n* = 6 for each treatment and tissue). **(B)** Quantification intensity and coherence of collagen. No significant differences were found before and after treatments (*n* = 6 for each) and in the two different portions. Mean values and ±standard deviation were considered. *p-value <0.05, **p-value < 0.01, ***p-value < 0.001, ****p-value < 0.0001. **(C)** SEM analysis. The cells visible in the native tissue, adipocytes and enterobacteria (in the smooth, a), and quiescent fibrocytes (in the wrinkled, c) were no more visible in the tissue decellularized with 15 S 9 (smooth, i; wrinkled, k) while they were still present in the wrinkled tissue after X 100 treatment (g) but not in the smooth (e). The native architecture of the ECM (smooth, b; wrinkled, c), characterized by bundles of type I collagen fibers and type IV collagen reticular fibers, did not appear changed after X 100 treatment (smooth, e, f; wrinkled, g, h). In contrast, after the use of 15 S 9, the collagen I fibers appeared divided into first- and second-order bundles (smooth i, j; wrinkled k, l). Scale bar: 5 and 10 m. SEM: scanning electron microscope. **(D)** TEM analysis. Plasma cells (smooth, a) and fibrocytes (wrinkled, c) were present in the native, while nuclear residues were confirmed after X 100 (smooth, e; wrinkled, g), while elastin was still visible in the fibers with a disorganized pattern (smooth, f; wrinkled, h). Absent nuclear residues after treatment with 15 S 9 (smooth, i; wrinkled, k) but with partial loosening between the organized fibers (smooth, j; wrinkled, l). Scale bar: 1 and 2 μm; 100 and 500 nm. TEM: transmission electron microscopy. (*n* = 6 for each).

In Figure 3A, the SHG signal (in white) (smooth [a–j]; wrinkled, [c–l]) represents the structure of collagen fibers (mainly collagen type I and IV). The fibrillar morphology of collagen, in smooth [a] and wrinkled [c] native porcine tissue, appeared as a dense and compact arrangement of undulating fibrillar structures (apparently thinner in the wrinkled tissue) with a spatial and multidirectional organization. This was confirmed by the similar distribution of DAPI-stained nuclei in the two portions of native tissue (smooth, [a] and wrinkled, [c]). No changes were detected in the fibrillar architecture after the use of X 100 (smooth, [e]; wrinkled, [g]) and 15 S 9 (smooth, [i]; wrinkled, [k]), which maintained a structure like the original one, without substantial damage of the ultrastructure. In this scenario, the absence of nuclei was also confirmed. Combining the 2D-FFT images with the SHG signal is possible to study the ECM structure of the tissue distinguishing collagen fiber bundles, with the same alignment, in the tissue ultrastructure, resulting in an elliptical or circular pattern with unaligned fibers. As shown earlier, an alignment in the fibrillar pattern of collagen was evident in the tissue only after decellularization (Figure 3A). Consequently, the representation in the 2D-FFT diagram showed a multidirectional orientation of the native fibers (pronounced in smooth, [b] limited in wrinkled, [d]) getting more unidirectional in the decellularized tissues after X 100 (smooth, [f]; wrinkled, [h]) and 15 S 9 (smooth, [j]; wrinkled, [l]) treatments.

These data were confirmed not only by the quantification of the structure of the fibrillar architecture, using the SHG Intensity signal (Collagen Intensity SHG) (Figure 3B), but also by the estimation of the collagen fibers (Collagen Coherency SHG) through SHG signal coherence analysis, before and after the use of the two different detergents (Figure 3B). Specifically, looking at the collagen intensity, no significant changes were present in the overall collagen architecture, of the SIS scaffolds, after decellularization treatments (Figure 3B). Moreover, evaluating the collagen coherency, (Collagen Coherency SHG) the fibers showed a better alignment, although not significantly, after decellularization with X 100 detergent, in both smooth and wrinkled portions of the tissue (Figure 3B).

SEM examination of the native porcine submucosa, in its smooth and wrinkled parts, showed a typical connective tissue ECM with intermediate characteristics of lax and compact (Figure 3C [a-f] smooth; [g-l] wrinkled). In the smooth and wrinkled parts, there were adipocyte [a] and enterobacterial cells [a], characteristic of the intestinal zone, totally incorporated in the ECM, and in the wrinkled part, quiescent fibrocytes [g] aligned parallel to the collagen bundles and with elongations embedded in the interstices of the fibrillar component, indicating an unstressed microenvironment.

The native structural pattern ([d], smooth; [j], wrinkled) of the intestinal submucosa showed a massive presence of collagens, both fibrillar (types I and III) and reticular types (mainly type IV). The structural pattern was composed of several bundles of second-order fibers, often forming a single large bundle, which flowed into and out of a dense network of fine fibers organized in a network. This architecture was similar in the smooth native portions [a, d] and in the wrinkled native portions [g, j], although, in the latter, the second-order collagen bundles had a more undulatory pattern.

After decellularization with X 100, no changes in the architecture were observed in the smooth [b, e], while in the wrinkled, there was a partial dismemberment of the fibers [h] and a persistent presence of cells [k], demonstrating a lower decellularization efficiency. The smooth and wrinkled collagen fibers, especially the second-order fibers, were not organized into one large bundle after the use of 15 S 9 but appeared dismembered into the first-order fibers (smooth [c, f]; wrinkled [i, l]). In contrast to the use of X 100, after the use of 15 S 9, the absence of cells and bacteria in the smooth and rugose areas was confirmed.

The presence of various specific cell populations in the porcine intestinal submucosa was also visible by TEM (Figure 3D). In the native, nuclei of plasma cells and macrophages in the smooth [a] and fibrocytes in the wrinkled part [g] were visible within the amorphous ECM substance together with a massive presence of collagen. Dense fibrils (visible in the smooth part, [d]), characterized by a regular band, a fibrillar diameter of 60–70 nm (visible in the wrinkled part [j]), and a limited presence of elastin (visible in the smooth, [d]), could be distinguished.

In both (smooth [a–f] and wrinkled [g–l]) portions, the ECM showed bundles of collagen fibers in a complex pattern throughout the matrix. The bundles’ orientation was parallel and perpendicular to each other. This arrangement offers considerable pressure and tensile strength and had not changed substantially after the treatments. A lower decellularizing efficacy of X 100, compared to 15 S 9, was confirmed, as membranous cellular remnants were still visible, in the smooth and wrinkled part of the submucosa (smooth, [b]; wrinkled, [h]). However, the integrity of collagen and elastin fibers was preserved (smooth, [e]; wrinkled, [k]). The improved efficacy of Tergitol 15 S 9 was further confirmed by the absence of cells or membranous residues, highlighted by the circular cavity left by them (wrinkled, [i]). There was also a partial loosening of the collagen fibers from the main bundle, while preserving the overall structure for both portions. Elastin was not visible (smooth [c, f]; wrinkled [i, l]).

A biochemical profile of smooth and wrinkled SIS portions after decellularization, with X 100 and 15 S 9, was evaluated. Specifically, the major protein contents, glycosaminoglycans (GAGs), elastin, and hydroxyproline (HYP), of porcine SIS were quantified after both decellularization protocols (Supplementary Figure S4). The amount of glycosaminoglycans (Supplementary Figure S4A) was unchanged in the smooth tissue after the decellularization treatments, whereas it was significantly decreased in the wrinkled tissue after treatment with X 100 (*p value = 0.0497) compared with the native one. Moreover, Supplementary Figure S4 shows that neither the elastin (b) nor the hydroxyproline (c) content, which is the indirect estimation of collagen, in the two smooth and wrinkled portions, were affected by the decellularization treatments with X 100 and 15 S 9.

Finally, the thermal characteristics of smooth and wrinkled SIS-dECM scaffolds were also evaluated. DSC was performed to determine the collagen contraction temperature, characterized by an endothermic peak (Supplementary Figure S5). The peak temperature was detected at 67.23 ± 0.08 and 67.31 ± 0.46°C wrinkled SIS, respectively; at 67.53 ± 0.37 and 67.53 ± 0.37°C for smooth and wrinkled decellularized SIS X100; at 68.25 ± 0.04 and 67.04 ± 0.15°C for smooth and wrinkled decellularized SIS 15 S 9 (Table 1). There were no significant differences between untreated (native) and decellularized SIS with X100 and 15 S 9 in both smooth and wrinkled SIS portions.

**TABLE 1:** Shrinkage temperatures detected for the investigated samples.

Samples	Shrinkage temperature [°c]
*Native*	
Smooth	67.23 ± 0.08
Wrinkled	67.31 ± 0.46
*X100*	
Smooth	67.53 ± 0.37
Wrinkled	67.54 ± 0.38
*15S9*	
Smooth	68.25 ± 0.04
Wrinkled	67.04 ± 0.15

### 3.5 Biomechanical assessment of smooth and wrinkled SIS dECM after decellularization

**FIGURE 4:**
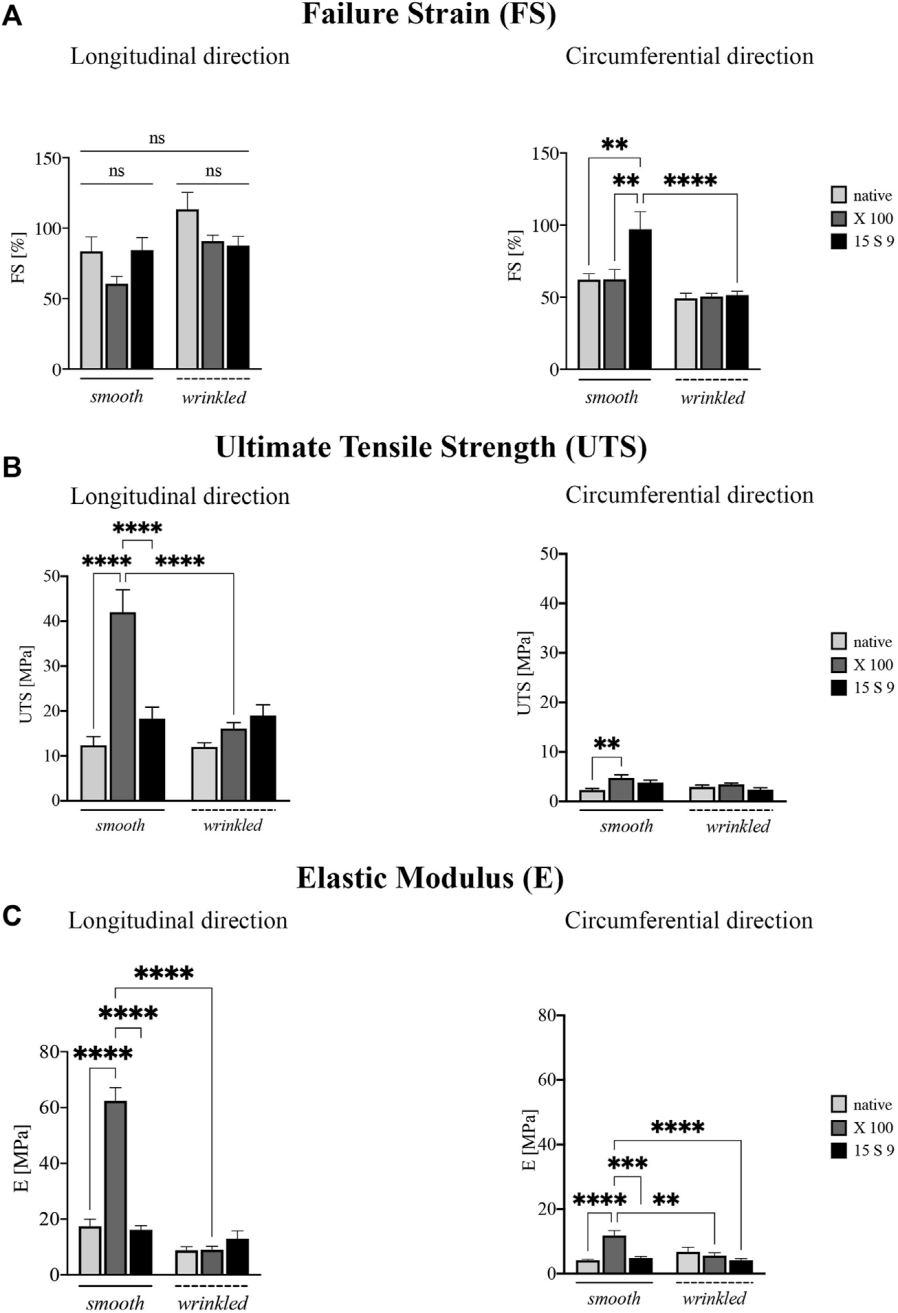
Biomechanical behavior of the SIS dECM along the longitudinal and circumferential directions. A preserved anisotropy, along the two directions and in the two portions of SIS (*n* = 3 for each), was present in all parameter values considered before and after the treatments. **(A)** Failure strain (FS). Failure strain values along the longitudinal direction were unchanged, while in the circumferential direction, the values after 15 S 9 were significantly increased, in the smooth compared to the native smooth (**p-value = 0.0035), with X 100 smooth (**p-value = 0.0040) and with 15 S 9 wrinkled (*p-*value <0.0001). **(B)** Ultimate tensile strength (UTS). X 100 decellularization tended to increase UTS values in the smooth along the longitudinal direction significantly compared to native smooth, 15 S 9 smooth and X 100 wrinkled (****p-value < 0.0001). In contrast, along the circumferential direction, the values were very low but significantly increased in smooth X 100 compared to native smooth (**p = 0.026). **(C)** Elastic modulus (E). The use of X 100 in the smooth longitudinal portion significantly increased the stiffness values (****p-value < 0.0001), compared to the native smooth 15 S 9 and after X 100 wrinkled (****p-value < 0.0001). Also, along the circumferential, the stiffness values of smooth X 100 were increased compared to native smooth and 15 S 9 wrinkled (****p-value < 0.0001) and compared to smooth 15 S 9 (***p-value = 0.0002) and wrinkled X 100 (**p-value = 0.0012). The parameter values are considered (FS, UTS, and E) as mean ± standard deviation, for each treatment, in the smooth and wrinkled portions and along the longitudinal and circumferential directions. X 100: Triton X 100.15 S 9: Tergitol 15 S 9.

The uniaxial tensile assessment of all tissue samples was performed along two directions, longitudinal and circumferential, to evaluate the possible anisotropy. The following parameters were calculated: failure Strain (FS) (Figure 4A, longitudinal and circumferential, respectively), ultimate tensile strength (UTS) (Figure 4B, longitudinal and circumferential, respectively), and elastic modulus (E) (Figure 4C, longitudinal and circumferential, respectively). The two portions of SIS, smooth and wrinkled, were considered before and after the decellularization treatments.

FS analysis showed values along the longitudinal direction (Figure 4A), not statistically different, in the smooth and wrinkled samples, after decellularization treatments, compared with native counterparts. Along the circumferential direction, a significant increase was present in 15 S 9 smooth compared to native (**p value = 0.0035), X 100 smooth (**p value = 0.0040) and 15 S 9 wrinkled (****p value < 0.0001). After decellularization with X 100, in smooth tissue, a significant increase in UTS was evident (Figure 4B). This increase was present along the longitudinal direction, comparing smooth native versus decellularized X 100 and comparing smooth X 100 versus smooth 15 S 9 portions (****p value <0.0001). There were no differences between native and decellularized wrinkled tissues. Smooth X 100 samples had a significantly increased longitudinal UTS compared to wrinkled X 100 (****p value <0.0001). Along the circumferential direction, there was a significant difference only between smooth native versus smooth X 100 tissues (**p value = 0.0026) (Figure 4B).

Similarly, the stiffness values (elastic modulus or Young’s modulus, E), presented in Figure 4C, were significantly increased in the smooth portion decellularized with X 100, both along the longitudinal and circumferential directions. Along the longitudinal direction, smooth native is significantly different compared to smooth X 100 and smooth 15 S 9 compared to smooth X 100 as well. Moreover, smooth and wrinkled X 100 (****p value <0.0001) are significantly different between them. Along the circumferential direction, native smooth is significantly different with respect to smooth X 100. Smooth X 100 is also significantly different when compared to smooth 15 S 9, wrinkled X 100, and wrinkled 15 S 9 (****p value < 0.0001; ***p value = 0.0002; **p value = 0.0012) (Figure 4C; Table 2).

**TABLE 2:** Biochemical properties of native and decellularized porcine smooth and wrinkled SIS.

Parameters	Longitudinal direction			Circumferential direction		
	Native	X100	15S9	Native	X100	15S9
Smooth						
Failure strain (FS)	83.51 ± 30.96	60.51 ± 16.08	84.43 ± 26.84	62.10 ± 12.48	62.48 ± 20.41	97.06 ± 36.52
Ultimate tensile strength (UTS)	12.33 ± 5.90	41.98 ± 15.18	18.28 ± 7.76	2.29 ± 0.97	4.74 ± 1.87	3.78 ± 1.56
Elastic modules (E)	17.43 ± 7.62	62.42 ± 14.25	16.10 ± 4.58	4.12 ± 0.92	11.78 ± 4.80	4.84 ± 1.63
Wrinkled						
Failure strain (FS)	113.47 ± 35.63	90.68 ± 12.90	87.51 ± 20.09	49.14 ± 10.76	50.37 ± 6.80	51.31 ± 8.25
Ultimate tensile strength (UTS)	11.94 ± 2.84	16.06 ± 4.13	19.01 ± 7.16	2.94 ± 1.09	3.42 ± 0.76	2.38 ± 1.15
Elastic modules (E)	8.76 ± 3.86	8.96 ± 3.76	12.93 ± 8.48	6.76 ± 4.41	5.58 ± 2.73	4.14 ± 1.78

These preliminary results showed a marked anisotropy of the tissues after both decellularization treatments due to the microstructural organization of the collagen fibers characteristic of the intestinal submucosa.

### 3.6 Biocompatibility assessment

The biocompatibility of medical devices for blood-contact applications is a prerequisite for their success, and the first step is the endothelization process. For any biomaterial that has to serve as a vascular graft, it is fundamental to assess the ability to properly interact with endothelial cells promoting their surface adhesion. Therefore, the growth and functions of HUVECs were evaluated to study their behavior on SIS-dECM treated with X 100 and 15 S 9.

**FIGURE 5:**
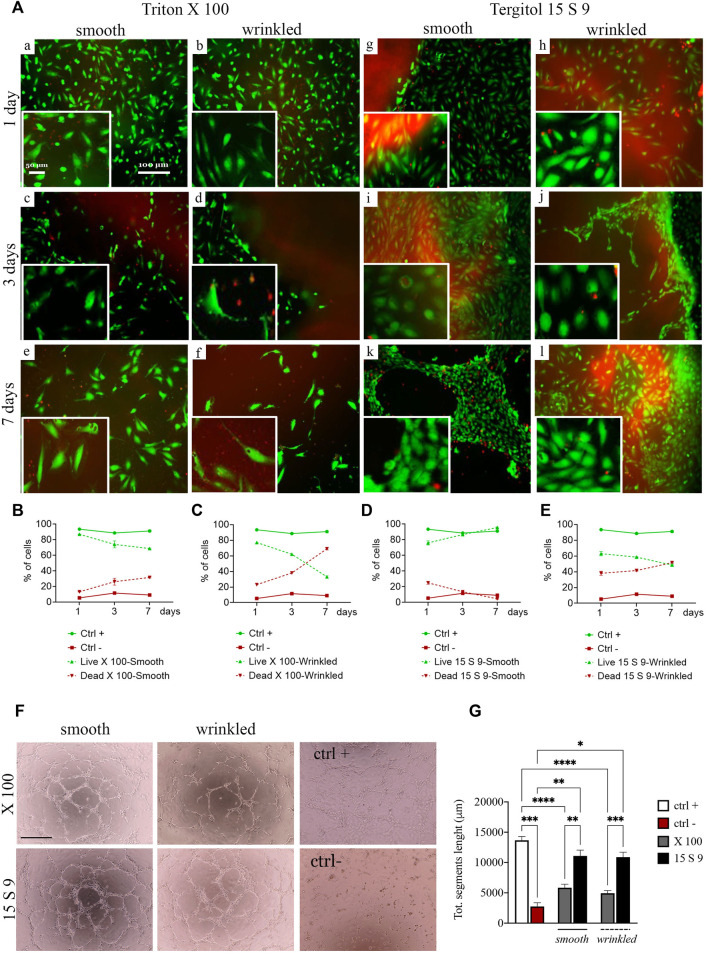
Cytocompatibility and angiogenic capacity retained after treatments. **(A)** Live/dead staining and quantification of SIS dECM. A decrease in viability from day 2 was observed in smooth (a, c, e) and wrinkled (b, d, f) tissue treated with X100 for all timepoints (1, 3, and 7 days). This is demonstrated by the quantification of HUVEC cell viability/mortality at different times **(B)**. In the wrinkled X100, mortality increased to a greater extent, starting from day 2, and more rapidly, as presented in the graph **(C)**. At no time did the HUVECs reach complete maturation and viability. In contrast, the cells of the 15 S 9 tissue found an excellent microenvironment. In fact, after 7 days, the HUVECs in the smooth tissue alone had organized themselves to form tubular structures characteristic of endothelial cells, values confirmed in the graph **(D)**. In contrast, in the wrinkled tissue treated with 15 S 9, there was a decrease in viability from day 2 **(E)**. **(F)** Tube assay. The formation of a thick, tight mesh network comparable to the positive control (ctrl +) was obtained globally with the smooth tissue treated with 15 S 9, while in the wrinkled, it was well formed but had some interruptions. In the wrinkled X 100, it appeared even more interrupted, while in the smooth X 100, a good mesh was present, but the meshes were not as tight and connected as in the smooth 15 S 9. In fact, in the graph **(G),** there is the quantification of the bond length of the HUVECs, there is a significant increase in the smooth 15 S 9 compared to the smooth X 100 (**p-value = 0.0034), and the wrinkled 15 S 9 also increased significantly compared to the wrinkled X 100 (***p-value = 0.0004). Scale bar: 100 μm. X 100: Triton X 100.15 S 9: Tergitol 15 S 9.

We performed live and dead cytocompatibility tests after decellularization with Triton X 100 (Figure 5A). After treatment with X 100 (Figure 5A [a-f]), cell viability was estimated to be 86% in smooth tissue and 77% in wrinkled tissue after 1 day, but after 7 days of culture, in both tissues, the percentage dropped to 68% and 32%, respectively (Figures 5B,C). The endothelial cells were less abundant and their shape was almost rounded and immature until the last day (Figure 5A, [a,c,e] smooth and [b,d,f] wrinkled), suggesting that the surface of the SIS dECM, so treated, was unsuitable for cell adhesion and diffusion.

The viability after decellularization with 15 S 9 (Figure 5A [g–l]) in smooth and wrinkled porcine tissues showed good cell viability, which increased rapidly at different time points with exceptional cell maturity in smooth tissue. Specifically, at day 1, in smooth and wrinkled tissues decellularized with 15 S 9 ([g], smooth and [h], wrinkled), the percentage of infiltrated cells was 78% and 61%, respectively (Figures 5D,E). After 3 days, in the smooth tissue, the cells were completely distributed in the smooth surface [i], entering only at the contact edge with the wrinkled tissue [j]. After 7 days, only in the smooth tissue [k], cells (97% compared with 49% of the wrinkled tissue) were organized to form a tubule, which is the characteristic conformation of HUVECs, indicative of a microenvironment with excellent biological properties. Cells appeared packed and completely in contact with each other, but only in the smooth tissues tubules were present.

In addition, a tube assay was performed to test the potential influence of decellularization treatments on the angiogenic ability of checking HUVECs’ capacity to form tubes and networks (Figure 5F). The difficulty of the cells to form stable cross-links, in the wrinkled tissue compared to the smooth tissue, was evident due to the frequently broken links, as occurred in the negative control. This was evident in the wrinkled tissue treated with X 100 rather than in the 15 S 9 one, where cross-links were broken but formed. In the smooth 15 S 9 portion, network formation was comparable to the positive control: the tubules appeared thicker and the number of cells creating the network was greater, resulting in a tighter mesh, which was evident in the smooth tissue after both 15 S 9 and X 100 detergents. These data were confirmed by the length values (μm) of the tubule segments formed by the cells, in contact with smooth and wrinkled tissues, decellularized with X 100 and 15 S 9 (Figure 5G). The length of segments, in smooth and wrinkled X 100-tissues, decreased significantly compared to the positive control (****p value<0.0001). No significant differences were detected compared with negative control. On the contrary, no significant differences were observed between positive control and 15 S 9 smooth and wrinkled tissues. Additionally, 15 S 9 treatment showed more angiogenetic features compared with X 100 treated tissues (smooth **p value = 0.0096 and wrinkled ***p value = 0.0004).

### 3.7 Proteomic content evaluation of treated SIS portions

**FIGURE 6:**
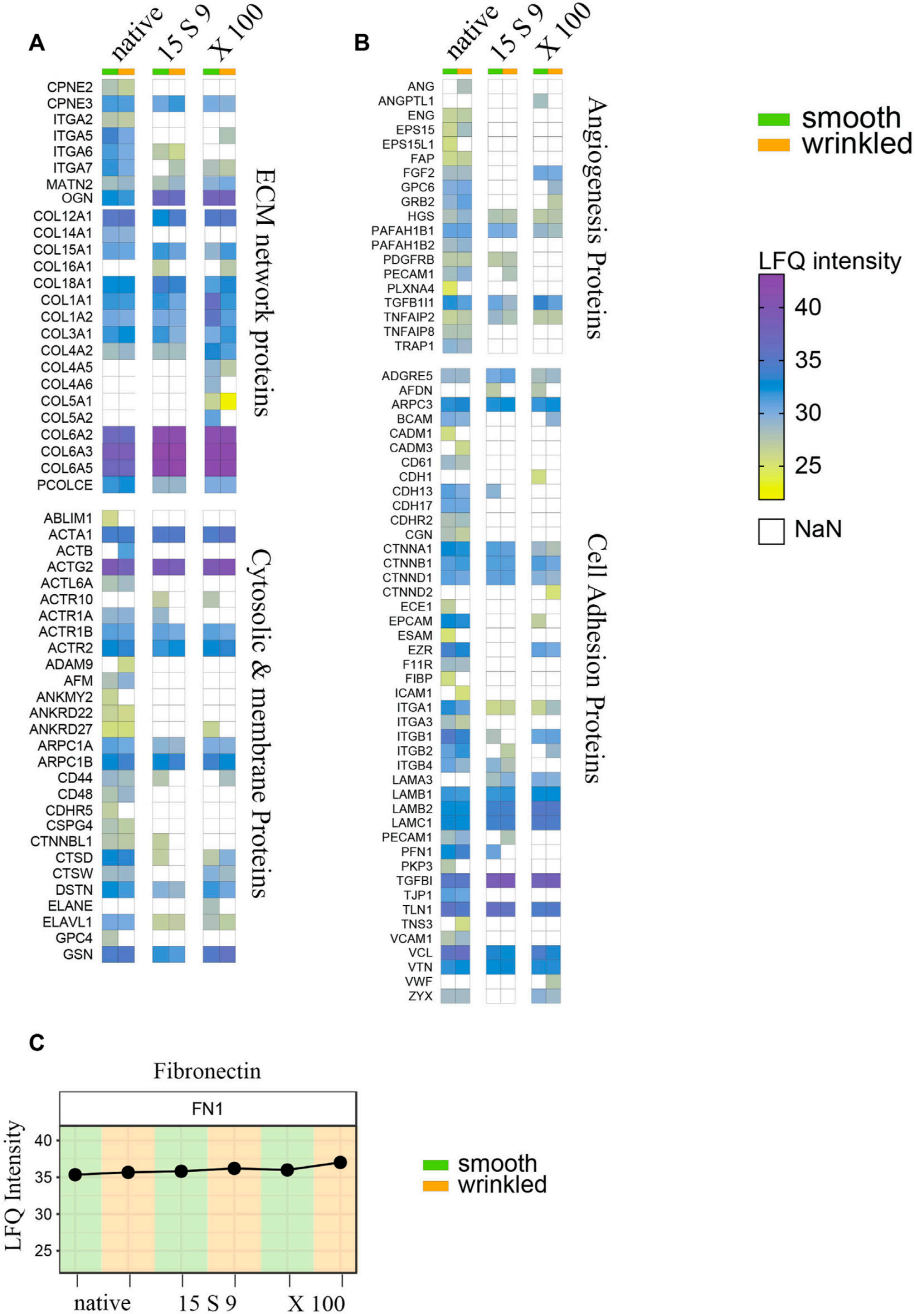
Full proteome analyses after treatments. Heatmap of label-free quantitation (LFQ) intensities of significantly changed proteins within smooth and wrinkled tissues, after both decellularization treatments, with the GO terms: **(A)** cellular proteins and collagen; **(B)** angiogenesis proteins and cell adhesion proteins. **(C)** Profile plots showing the Log_2_ LFQ intensity for the fibronectin protein detected in treated SIS tissues.

From proteomic results, three basic categories, for tissue remodeling, can be considered: 1) the composition of ECM proteins, with the abundant amount of collagen and fibronectin and a subgroup of cytosolic and membrane proteins, 2) proteins that directly or indirectly induce angiogenesis, and 3) cell adhesion (Figures 6A–C). The native composition of the ECM showed a variety of protein components related to the structure of the matrix itself and involved in signaling, in equal amounts in smooth and wrinkled portions.

Most of the cytosolic and membrane proteins were lost or significantly reduced after the decellularization treatments. Actin, several of its subunits, and components of the microfilaments were present in abundant amounts in the native tissue, but their peptides intensities were undetected or significantly lower in the decellularized samples. Several proteins related to the ECM network proteins group, such as copines (CPNE2 and CPN3) and integrins (ITGA2, ITGA5, ITGA6, and ITGA7), were decreased in both decellularizations. Matrillins (MATN2), mediating interactions between ECM components, like collagens and proteoglycans, were instead conserved after X 100 or 15 S 9 treatments.

Massively present and well preserved were collagen proteins, detected in both portions and perfectly preserved after both decellularizations, and fibronectin (Figures 6A,C). The more represented were the following: Col VI (COL6A2, COL6A3 and COL6A5), Col I (COLA1, COLA3), Col III (COL3A1), and Col IV (COL4A2). Some subtypes were also detected, such as Col XII (COL12A1), XVIII (COL18A1), and Col XV (COL15A1). Growth factors and molecules inducing angiogenesis were detected too, including FGF (FGF2), TGF-β (TGFB1), endoglin (ENG), platelet activators (PAFAH1B2, PAFAH1B1), and endothelial cell–specific (PECAM1) proteoglycans (GPC6), decreased after both decellularizations, in both portions, except FGF maintained after X 100 (Figure 6B). In the third group, we identified proteins that are directly or indirectly involved in the cell adhesion process. These include several adhesion proteins such as G-protein-coupled proteins (ADGRE5) expressed in many cell types, conserved structural and adhesive molecules, including catenin (CTNNA1, CTNNB1, and CTNND1), integrins (ITGA1, ITGA3, ITGB1, and ITGB2), laminins (LAMB1, LAMB2, and LAMC1), talin (TLN1), vinculin (VCL), vitronectin (VTN), and zixin (ZYX).

## 4 Discussion

The use of prosthetic patches, either biological or synthetic, to appropriately repair cardiac structural lesions in the operating room, is very frequent in cardiac surgery (Holubec 2014). Focusing our attention on this type of clinical scenario, the main objective of the study was to create, from heterotopic swine tissue, a decellularized ECM with preserved structure and bioinductive properties. The resulting scaffolds will be useful not only as biomaterials for pure surgical reconstruction but also as substrates for tissue remodeling. In this sense, recently, the small intestinal submucosa (SIS), a submucosa extracted from the jejunal portion of the pig’s small intestine, between the mucosal and muscular layers, has received much attention. This tissue was proved to be biocompatible, strong, and durable due to its structural properties and strong bioinductive properties that confer the scaffold’s important tissue remodeling capacity once grafted. SIS-ECM has been on the market for several years as Cormatrix© (CorMatrix Cardiovascular, Inc., Roswell, Atlanta and Alpharetta, GA, United States), as an acellular patch approved by the FDA (Food and Drug Administration) in 2010, for cardiac and extracardiac indications. While in preclinical studies, SIS-ECM has shown very encouraging results (Badylak et al., 1989; Lantz et al., 1992; Fallon, 2011; Padalino et al., 2012; Baker, 2018), the application of CorMatrix® in the clinic has not produced the same results. Immune reactions, inflammatory processes with the thickening of the patch, and finally calcifications in the surrounding tissue, were present in almost all cases considered (Woo, 2015; Nelson et al., 2016; Chakraborty 2021; Sood et al., 2021). To achieve our goal, we compared two decellularization protocols: the first based on the use of Triton X 100, as a non-ionic detergent and the second with its substitute, Tergitol 15 S 9. Briefly, our results show that SIS tissue, extracted from the proximal portion of the small intestine, is macroscopically formed into two regions: a smooth and a wrinkled portion. Both consist of connective tissue with intermediate properties of laxity and density. The overall analysis showed that the structure (Figure 2), ultrastructure (Figure 3), biochemical (Supplementary Figure S4), proteomic (Figure 6), and thermal (Supplementary Figure S5) profiles of the two regions of SIS are equivalent. The only differences were tissue anisotropy, the mechanical behavior along the longitudinal and circumferential directions (Figure 4), native cell population (Figure 1), and thickness (Supplementary Figure S6D). Native cell quantity and thickness were found to be greater in the wrinkled, while the cellular proneness to repopulation, migration, and communication was better on the smooth surface than in the wrinkled (Figure 5).

In more detail, histological analysis and DNA quantification demonstrated a decellularization efficacy of Tergitol 15 S 9 that was equivalent to Triton X 100. The benchmarks, like the amount of DNA, found to be less than 50 ng dsDNA/mg ECM dry weight (Figure 2A), and a complete absence of nuclei, were largely achieved (Figure 2B) (Crapo et al., 2011; Gilbert et al., 2006). These data indicate a good nuclear removal efficiency, in the case of Triton X 100, already described in the literature (Spina et al., 2003; Wang et al., 2017; Shahraki et al., 2022) and validated by the decrease in biomaterial thickness, after both treatments (Supplementary Figure S6D). The combination of non-ionic detergents, such as Triton X 100 or Tergitol 15 S 9, with bile acid adjuvants, seems to be successful, as it can give excellent results in terms of the amount of final DNA even at low detergent concentrations (Oliveira et al., 2013; Paniagua Gutierrez et al., 2015). The ECM is the fundamental unit of any tissue and it is influenced by the microenvironment into which it is integrated. In response to the changing environment, the ECM adapts in a process of ‘dynamic reciprocity’ (Labat-Robert et al., 1990). Indeed, ECM has been shown to help cells communicate and differentiate among themselves (Badylak, 2007). In this scenario, the extent of capillary growth and cell migration depends on the architecture in which they are located. Therefore, after decellularization, in the absence of the original cells and their signals, the architecture and ultrastructure that the ECM assumes become crucial, as it can guide cell repopulation and tissue remodeling, *in-vivo* (Bissell and Glennhall, 1982; Labat-Robert et al., 1990; Almarza et al., 2008). Of note, we did not observe alterations in the overall collagen fibers architecture and fibrillar directionality, by TPM microscopy (Figures 3A,B), which was assessed after both decellularization treatments, in contrast to what has been described in the literature for decellularized SIS (Casarin et al., 2022). Although the efficacy of the two detergents seems similar, in SEM and TEM microscopy analysis (Figures 3C,D), the validity of Tergitol 15 S 9 is better as no cells or membranous remnants are visible in the ultrastructure. Despite this, the fibers appear slightly thinned in the SEM after Tergitol 15 S 9. This is probably caused by more effective decellularization, but without causing the ECM to collapse, which instead retained a perfect 3D structure, unlike other treatments (Janis et al., 2012). In contrast, after the use of Triton X 100, the collagen fibers appeared disrupted and disordered (visible at TEM), probably due to significant protein denaturation (Lee et al., 2019).

The mechanical behavior of the SIS (Figure 4) was assessed along the two longitudinal and circumferential directions by uniaxial tensile test. Several studies report a reduction in mechanical strength after decellularization and freeze-drying treatments, including those with Triton X 100 (Syed et al., 2014). Our preliminary results show that stress along the (longitudinal) direction of the tissue treated with Triton X 100 straightened the wavy collagen fibers of the smooth portion, significantly increasing the values of resistance (UTS) and stiffness (E) (Figures 4A–C). The explanation could be found in the mechanism of action of Triton X 100 (Lee et al., 2019): due to its chemistry, the alkyl tail could have induced a significant protein denaturation that modified the collagen fibers, as shown by TEM images. In contrast, the use of Tergitol 15 S 9 did not modify the mechanics of the tissue, which maintained the values of the same parameters considered, comparable to the native tissue. Once again, these preliminary data confirm the superior preservation of the collagen fibers.

The longitudinal direction is where the values, of the considered parameters, are significantly greater than those measured in the circumferential counterpart (UTS and E). This characteristic of anisotropy remained unchanged after each treatment. The reason is that the organization of the fibers forms a biaxial angle, consisting of two populations of collagen fibers of approximately +30° and −30° along the longitudinal direction, which is therefore the preferred direction. Typical of this compartment are movements of retraction and dilation of the small intestine, during the transport of food along the digestive lumen (Orberg et al., 1983; Hiltner et al., 1985; Sacks and Gloeckner, 1999).

Key proteins of the SIS-ECM, such as hydroxyproline, elastin, and GAGs, (Supplementary Figure S4), remained unchanged after treatments in both regions. GAGs are crucial as their presence is associated with growth factors. GAGs are polyanionic molecules with a Na^2+^ net charge. This net charge attracts water, causing the interstitial spaces in which they reside to swell. This swelling can regulate the diffusion of many growth factors, promoting cell invasion and migration (Rozario and DeSimone, 2010). Although decellularization can induce a decrease in GAGs content (Courtman et al., 1994; Choe et al., 2018), our results showed that the amount of these GAGs was unaffected (Supplementary Figure S4A). Further supporting these results, the thermal profile (Supplementary Figure S5) also did not change after the X 100 and 15 S 9 treatments. The temperature of collagen denaturation in SIS tissues was similar after both decellularizations and in the smooth and wrinkled portions. Moreover, following disinfection with our protocol, all intestinal tissues decellularized with Triton X 100 and Tergitol 15 S 9 were sterile (Supplementary Figures S1, S2).

Excellent biocompatibility is critical for *in-vivo* implantation and the ability to support new blood vessel growth, which is necessary for constructive remodeling. Ideally, the graft surface must support rapid endothelialization, while minimizing platelet adhesion and subsequent thrombus formation (Prevel et al., 1994). It is already known that SIS is an exceptional substrate for human umbilical vein endothelial cell (HUVEC) adhesion and proliferation (Badylak et al., 1999; Woods et al., 2004; Quillard et al., 2010). This is due to the natural presence of fibronectin (FN) and collagens. Both have a specific RGD peptide sequence consisting of arginine (R), glycine (G), and aspartic acid (D) that is responsible for the recognition of the initial endothelial adhesion, in the SIS scaffold (Hodde et al., 2002). Following the literature, our results showed that the viability of human umbilical vein endothelial cells (HUVEC) on porcine, smooth, and wrinkled intestinal scaffolds, following the two decellularization protocols, was optimal, but greater after the use of Tergitol 15 S 9 and specifically in the smooth region (Figure 5). Indeed, the use of this detergent favored the formation of an excellent microenvironment here, allowing cells to organize in characteristic cellular models. We think that these promising results are also related to the ability of the cells in being able to come into contact and migrate on a smooth, obstacle-free surface versus a wrinkled one. In contrast to what has been described in the literature where the presence of pores is useful for the diffusion of the necessary oxygen and therefore better able to maintain cell viability (Androjna et al., 2008).

Proteomic analysis (Figure 6) showed the preservation of protein content in our biomaterials, after decellularization treatments. We focused on the structural and non-structural components of the ECM, the mediators of angiogenesis and those of cell adhesion, in the two portions and after the treatments. The complete set of proteins appeared slightly decreased in the two portions and equally after the two decellularization protocols, as already known (Ji et al., 2019). However, the main structural molecules and cell signaling mediators remained preserved in equal amounts in the two portions, after the treatments, indicating a similar decellularizing mechanism of action. The process of cell-ECM adhesion occurs in different, small, but largely specialized regions called plaques or focal adhesions. These structures are responsible for mediating the information coming from the ECM, influencing cell behavior. Molecules, such as talin 1, Zyx, and vinculin, play a key role in cell adhesion through complex mechanisms, providing cells with information on chemical and physical cues (stiffness and composition) as well as mechanical stimuli from the ECM (Nayal et al., 2004). In our experimental setting these proteins, are preserved. Especially, zyxin has an important role in the mechanotransduction of the adhesive membrane (Wang et al., 2019). Most collagens, and several subtypes, were preserved after the two decellularization processes and in the two portions. The non-collagen component was also present, such as fibronectin and proteoglycans. Indeed, enzymatic treatments with trypsin and acids damage key elements of the biomaterial (Abraham et al., 2000; Luo et al., 2011).

The vast presence of blood vessels, in SIS tissues, is indicative of the massive presence of pro-angiogenic growth factors, such as vascular endothelial cell growth factor (VEGF), which promotes angiogenesis, the permeability of endothelial cells, and stimulates their proliferation and migration or the transforming growth factor beta (TGF-β), which promotes cell proliferation and differentiation, wound healing and inhibits macrophage and lymphocyte proliferation (Hodde et al., 2001; Hurst and Bonner, 2001; Mcdevitt et al., 2003). Our results showed an absence of VEGF and a decrease in angiogenic factors, although growth factor like TGF-β 1 (TGFB1) was perfectly preserved after Tergitol 15 S 9. Several studies hypothesize a loss of angiogenic factors following the sterilization process (Abraham et al., 2000; Luo et al., 2011; Keane et al., 2012; Gaetani et al., 2018). A second hypothesis could be related to the lower angiogenic capacity of the intestinal area considered in the study, the proximal one. The small intestine is divided into two regions, the distal and the proximal. The former is the part 300–400 cm distal to the ileocecal valve, while the proximal is the part within the first 100 cm of the jejunum 50 cm from the duodenum (Ashley et al., 2010). In the study by Ashley et al., bladder patches of distal pig SIS resulted in improved regeneration of bladder tissue in rats following partial bladder removal (Ashley et al., 2010). Therefore, to validate these results, the tube formation test was performed, which is considered among the most reliable of the *in vitro* methods (DeCicco-Skinner et al., 2014; Kelley et al., 2022). Our results showed a preserved angiogenic capacity with clear pro-angiogenic superiority of Tergitol 15 S 9 over Triton X 100 and in the smooth than the wrinkled, confirming the better dynamics of endothelial cells in the smooth and homogeneous surface than in a porous and richly obstructed one. This was confirmed by the significant increase in segment length in the smooth after the use of Tergitol.

## 5 Conclusion

In conclusion, the use of Tergitol 15 S 9 in porcine tissues decellularization as a substitute for Triton X 100 has been proved in terms of higher decellularization efficacy. The tissue of SIS consists of two portions: smooth and wrinkled. The smooth surface seems to favor endothelial cell seeding and therefore tissue re-endothelialization and best performance once applied to the clinical practice. We have also shown that the bioscaffold resulting from decellularization with Tergitol 15 S 9 performs better due to a well-preserved 3D structure and ultrastructure, biochemical profile, and mechanical behavior. Finally, we have shown that with our protocols, SIS-derived matrices preserve their bio-inductive capacities, being able not only to create an excellent substrate for cells’ repopulation but also to favor new angiogenetic processes.

## 6 Limitations

It is worth mentioning some limitations of the present study. The major limitation is the thickness of the SIS tissue (0.100–0.160 mm), which is too thin to resist the pulsatile pressures and shear stress at the cardiovascular level. Therefore, techniques for increasing thickness, without altering the mechanical properties, should be considered for the applicability of the patch. The dynamics of bioinduction by SIS are not yet well understood, but the importance of retaining as many growth factors as possible is understandable. It would therefore be useful, for the purpose of tissue remodeling, to avoid the impairment of growth factors in SIS tissue during treatments, irrespective of the intestinal region considered. Furthermore, this study was performed using hearts obtained from a slaughterhouse; therefore, the sample numerosity was limited to three groups, for biomechanical tests, as the COVID-19 pandemic dramatically reduced access to the facilities and the resulting availability of tissues. Finally, *in vivo* tests will be crucial, on the one hand, to assess the response to constructive remodeling at different times and then to assess the presence of specific epitopes, after decellularization, which may be useful to predict an immune reaction *in vivo*, first of all by quantifying the presence of alpha-gal, which appears to be the greatest immune obstacle between man and the other mammalian species from which bioprostheses are obtained.

## Data Availability

The original contributions presented in the study are included in the article/Supplementary Material; further inquiries can be directed to the corresponding author.
