# Supplementary material for: Small intestinal submucosa-derived extracellular matrix as a heterotopic scaffold for cardiovascular applications
Source: Front Bioeng Biotechnol. 2022 Dec 12;10:1042434. doi: 10.3389/fbioe.2022.1042434 (PMC9792098; doi:10.3389/fbioe.2022.1042434)
Supplement: Supplementary file 1 [file DataSheet4.docx]

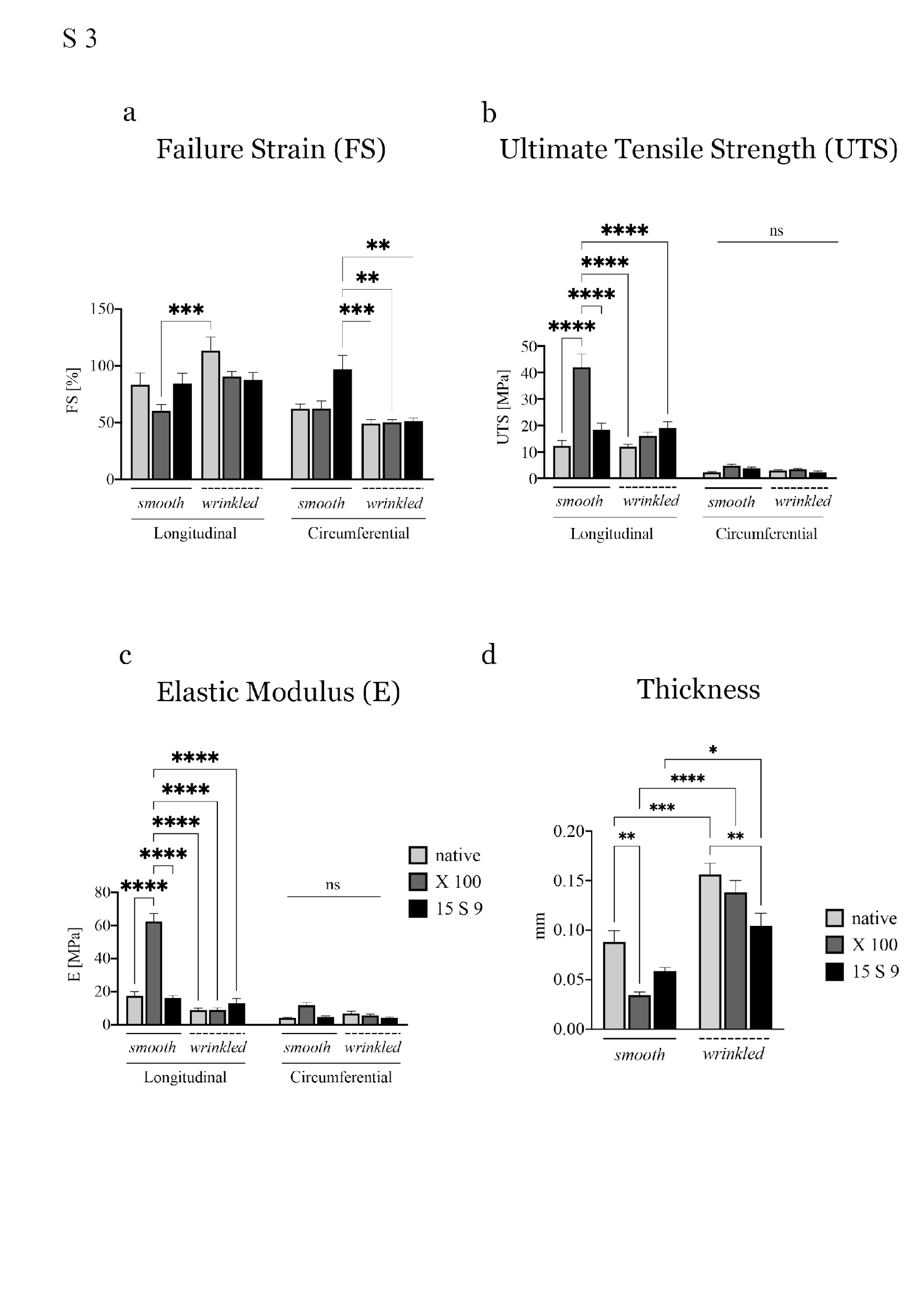


Supplementary Figure 6

Figure S6. Total biomechanical analysis and thickness of the SIS before and after treatment with X 100 and 15 S 9, along the longitudinal and circumferential directions. A reconfirmed tissue-typical anisotropy is maintained after each treatment. (a) Failure Strain (FS). A decrease in smooth X 100 compared to native wrinkled (***P value=0.0001), along the longitudinal direction. While in the circumferential direction a significant increase in smooth 15 S 9 was present compared to the native wrinkled side (*** p value=0.0008), X 100 (**p value= 0.0013) and after 15 S 9 (**p value=0.0018). (b) Ultimate Tensile Strength (UTS). In the longitudinal, a significant increase was present in the smooth X 100 compared to the native and 15 S 9 smooth and also in the native and 15 S 9 wrinkled (****p value<0.0001). Along the circumferential direction, however, no significance was registered. (c) Elastic Modulus (E). (d) Thickness. The thickness was significantly different between the two native regions, significantly greater in the rough (***p value=0.0002), compared to the smooth. After both treatments, it decreased significantly in X 100 smooth compared to native (p value 0.0048), X 100 wrinkled (p value< 0.0001). The wrinkled tissue retained higher values even though it decreased after the treatments, after 15 S 9 (**p value).

An increased stiffness was present after X 100 smooth in comparison to native and 15 S 9 smooth (****p value<0.0001) and also in comparison to wrinkled side, native, X 100 and 15 S 9 (****p value<0.0001). One-way ANOVA, Tukey's multiple comparison test, * p<0.05 **p 0.0021, *** p 0.0002, **** p <0.0001.

Supplementary Figure 7


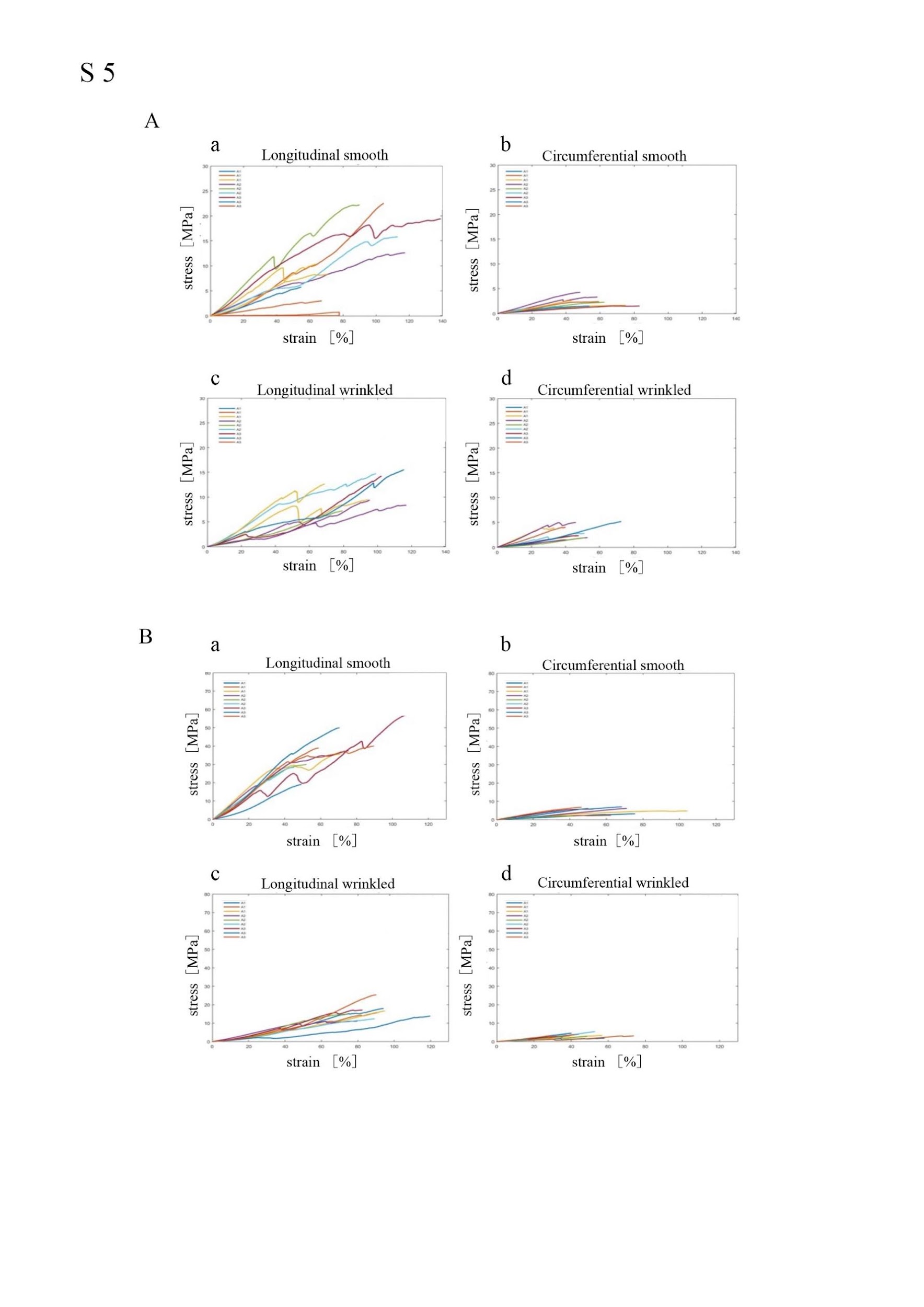


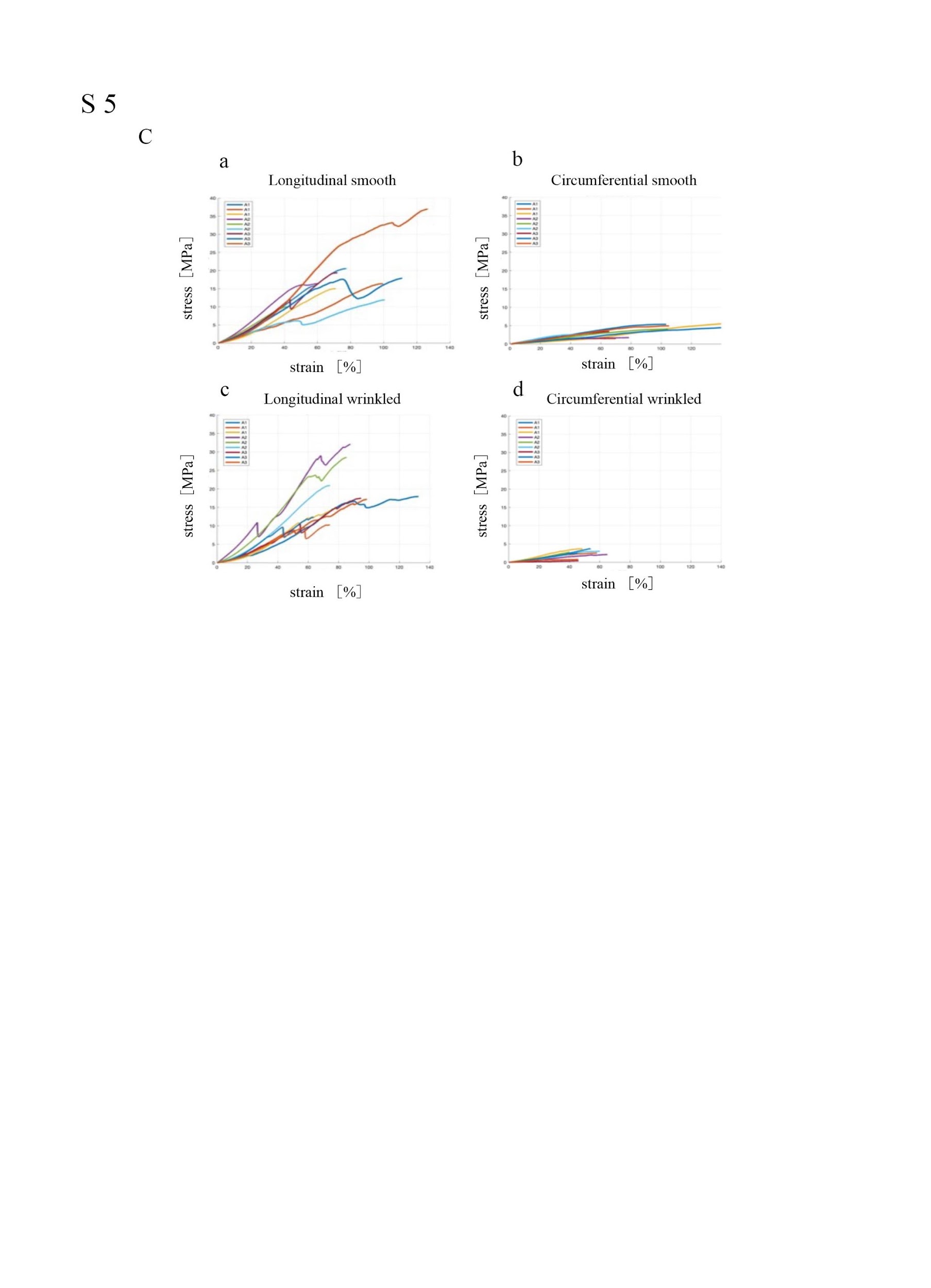


Figure S7. Biomechanical uniaxial tests. (A) Stress-strain curves of native SIS along longitudinal and circumferential direction in smooth and in wrinkled (respectively a, b and c, d). The characteristic anisotropy of the tissue is showed in the smooth portion, where the values of the parameters of interest are higher along the longitudinal direction than in the circumferential direction; (B) Stress-strain curves of decellularized SIS with Triton X 100 along longitudinal and circumferential direction in smooth and in wrinkled (respectively e, f and g, h). A difference in the parameters in the two directions is evident with a predominance along the longitudinal direction; (C) Stress-strain curves of decellularized SIS with Tergitol 15 S 9 along longitudinal and circumferential direction in smooth and in wrinkled (respectively A, B and C, D). Even after the use of Tergitol 15 S 9 the anisotropic characteristic remained with predominance along the longitudinal direction.
