# Supplementary material for: Small intestinal submucosa-derived extracellular matrix as a heterotopic scaffold for cardiovascular applications
Source: Front Bioeng Biotechnol. 2022 Dec 12;10:1042434. doi: 10.3389/fbioe.2022.1042434 (PMC9792098; doi:10.3389/fbioe.2022.1042434)
Supplement: Supplementary file 2 [file DataSheet3.docx]

Supplementary Figure 4
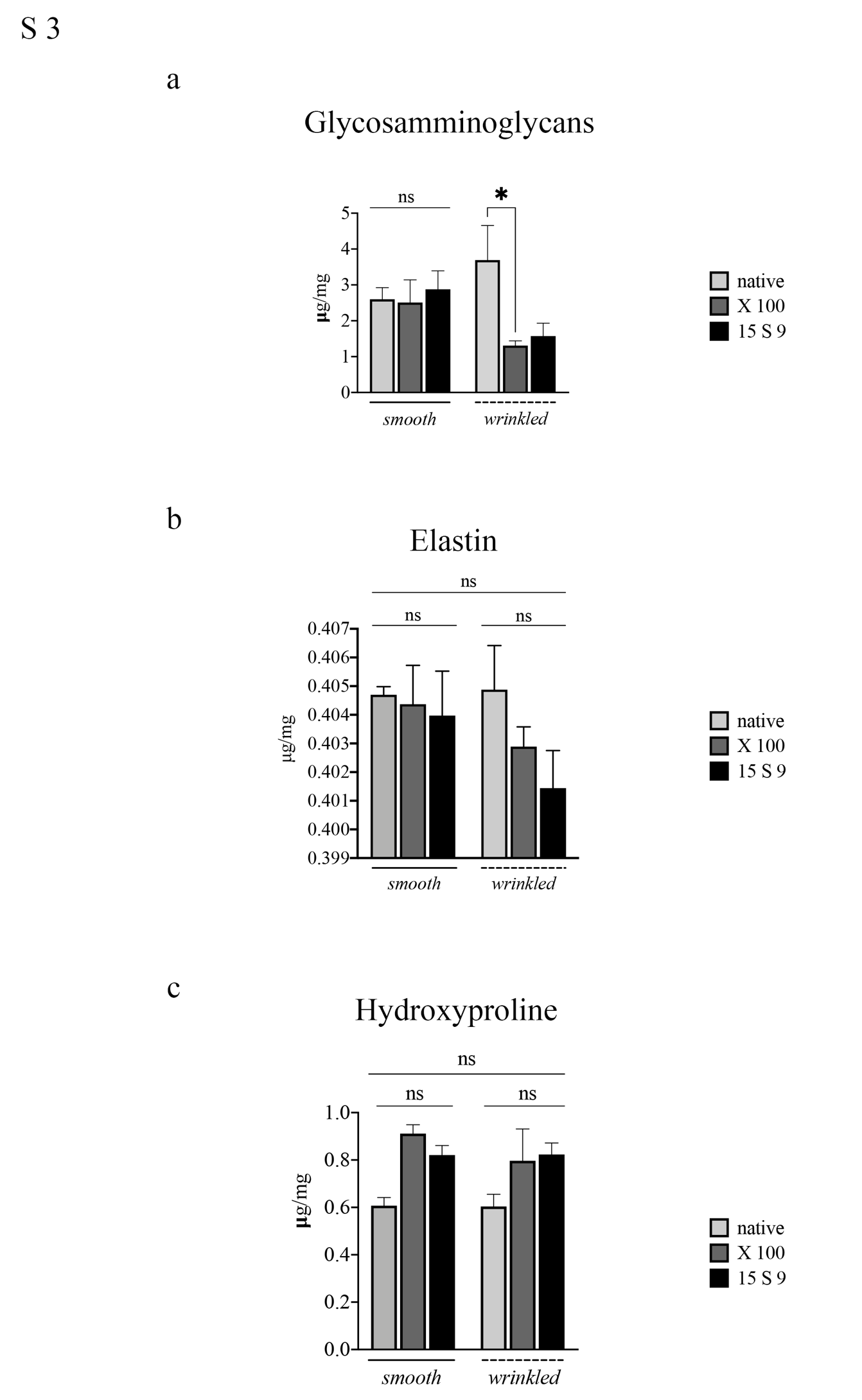


Figure S4. Quantitative biochemical profile evaluation of the main protein profile in SIS before and after decellularization treatment with the two different detergents. (a) Sulphated glycosaminoglycans (sGAG) was significantly low in wrinkled tissue treated with X 100 compared with native one; in (b) Elastin content and (c) Amount of Hydroxyproline (HYP) no statistically significant differences was detected, in the amount of the proteins considered, in both pig tissue before and after decellularizations. One-way ANOVA, Tukey's multiple comparisons test, * p< 0.05, **p 0.0021, *** p 0.0002, **** p <0.0001. X 100= Triton X 100; 15 S 9= Tergitol 15 S 9.

Supplementary Figure 5


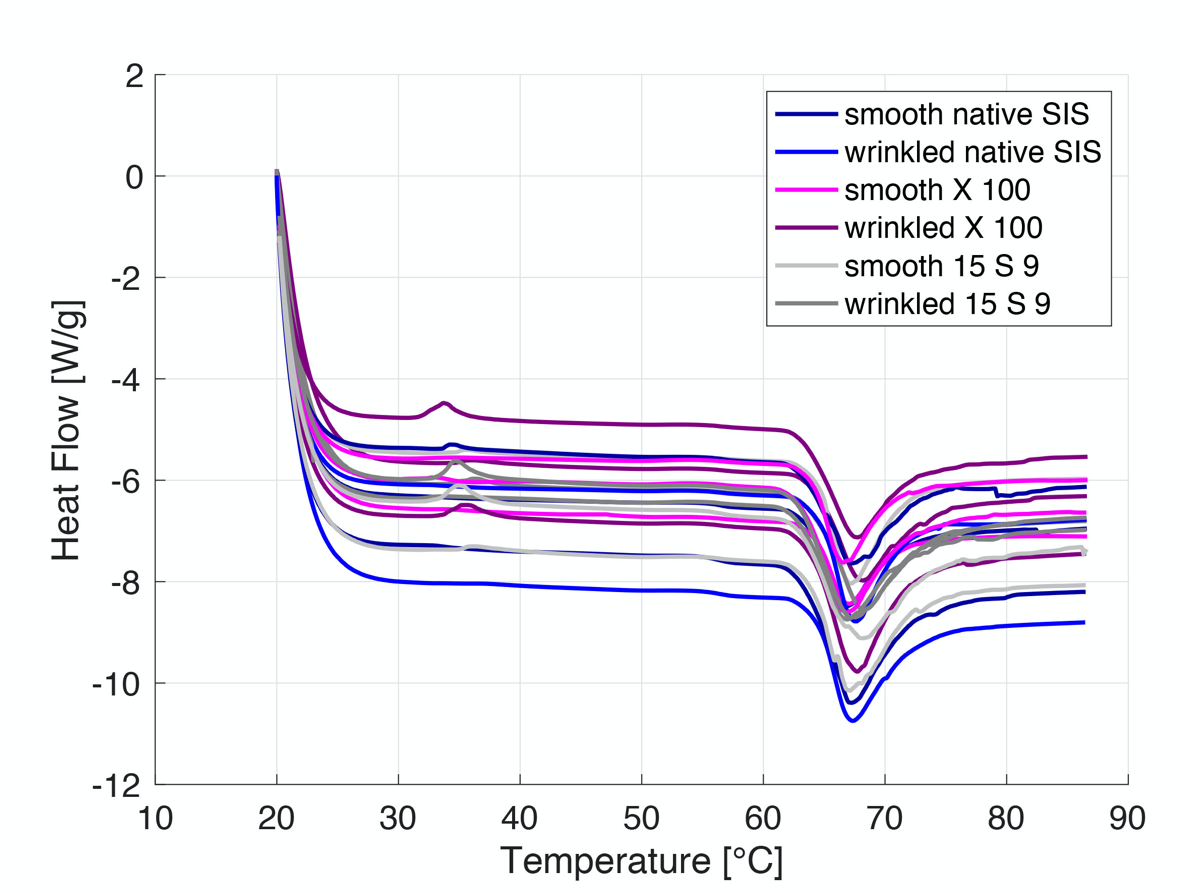


Figure S5. Experimental thermogram curves obtained from DSC analysis of the following tissues: native SIS, decellularized sis X100 and decellularized SIS 15 S 9, smooth and wrinkled. The temperature of collagen contraction (melting temperature, which is the temperature of collagen denaturation), characterized by an endothermic peak, revealed no significant differences between both native and decellularized SIS with X100 and 15 S 9, in both smooth and wrinkled SIS.

Specifically, the melting temperature was found to be 67.23 ± 0.08 and 67.31 ± 0.46 °C for native, smooth and wrinkled SIS, respectively; 67.53 ± 0.37 and 67.53 ± 0.37 °C for smooth and wrinkled decellularized X100 SIS; and 68.25 ± 0.04 and 67.04 ± 0.15 °C for smooth and wrinkled decellularized 15 S 9 SIS.
