# Supplementary material for: Small intestinal submucosa-derived extracellular matrix as a heterotopic scaffold for cardiovascular applications
Source: Front Bioeng Biotechnol. 2022 Dec 12;10:1042434. doi: 10.3389/fbioe.2022.1042434 (PMC9792098; doi:10.3389/fbioe.2022.1042434)
Supplement: Supplementary file 3 [file DataSheet2.docx]

Supplementary Figure 3


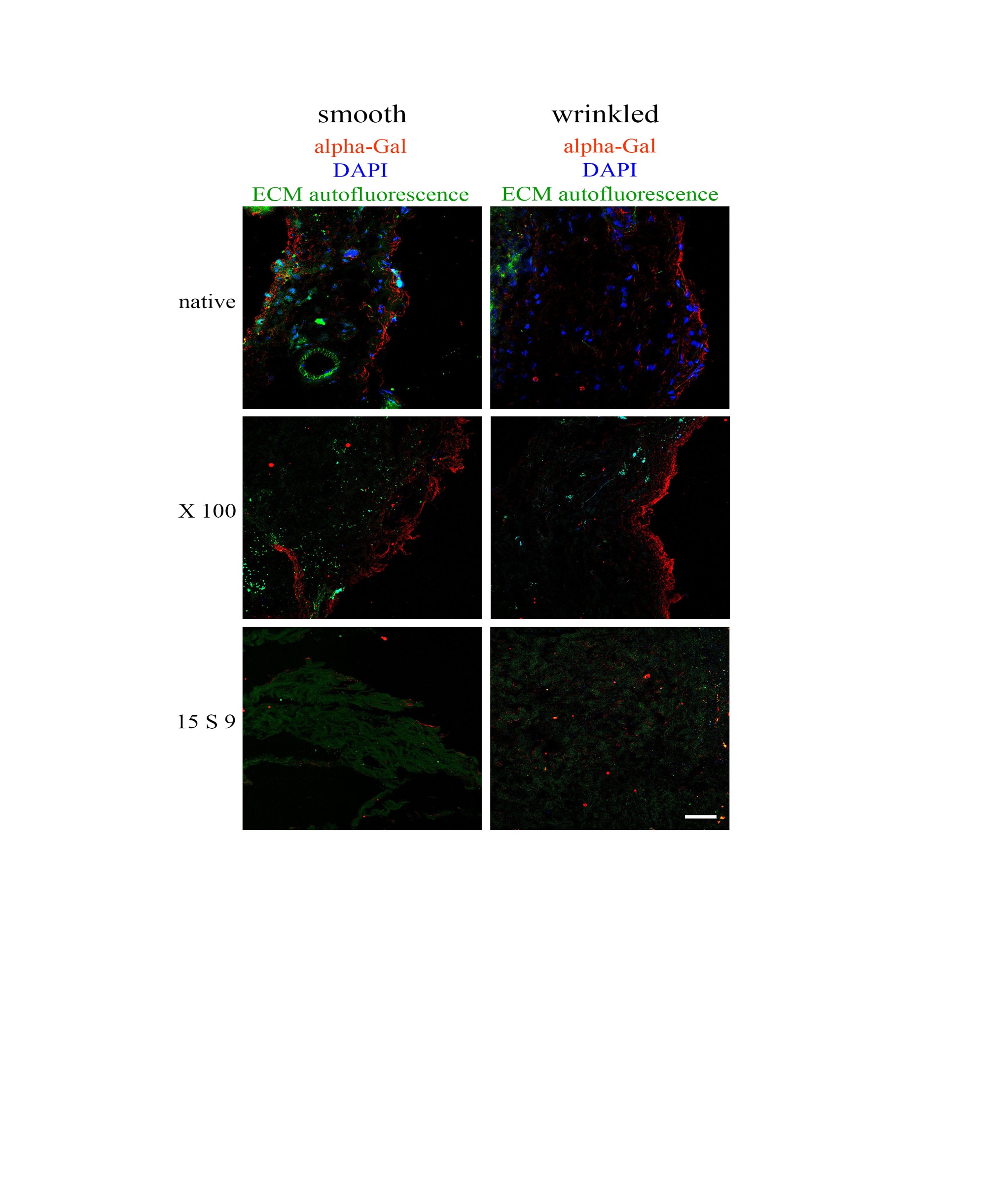


Figure S3. Immunofluorescence analysis of alpha-Gal epitope in native and SIS dECM. Comparison of smooth and wrinkled porcine SIS tissue, native and after decellularization with Triton X 100 and Tergitol 15 S 9 of the presence of alpha-Gal (in red).
The alpha-Gal epitope (in red) was present in large amounts in the native tissues, in equal amounts in the smooth and wrinkled, following a linear trend to be more expressed along the edges of the sample. DAPI-stained nuclei (blue) were present, apparently to a greater extent in the wrinkled than in the smooth. After decellularization X 100, the epitope was still massively present and followed the same course as the native. A few nuclei (blue) were still present, in the wrinkled mainly. In the 15 S 9 samples, on the other hand, the presence of alpha-Gal had decreased. No nuclei were present (blue). Scale bar 100 μm, magnification 40X SIS= small intestinal submucosa; X 100= Triton X 100; 15 S 9= Tergitol 15 S 9.
