# Supplementary material for: Small intestinal submucosa-derived extracellular matrix as a heterotopic scaffold for cardiovascular applications
Source: Front Bioeng Biotechnol. 2022 Dec 12;10:1042434. doi: 10.3389/fbioe.2022.1042434 (PMC9792098; doi:10.3389/fbioe.2022.1042434)
Supplement: Supplementary file 4 [file DataSheet1.docx]

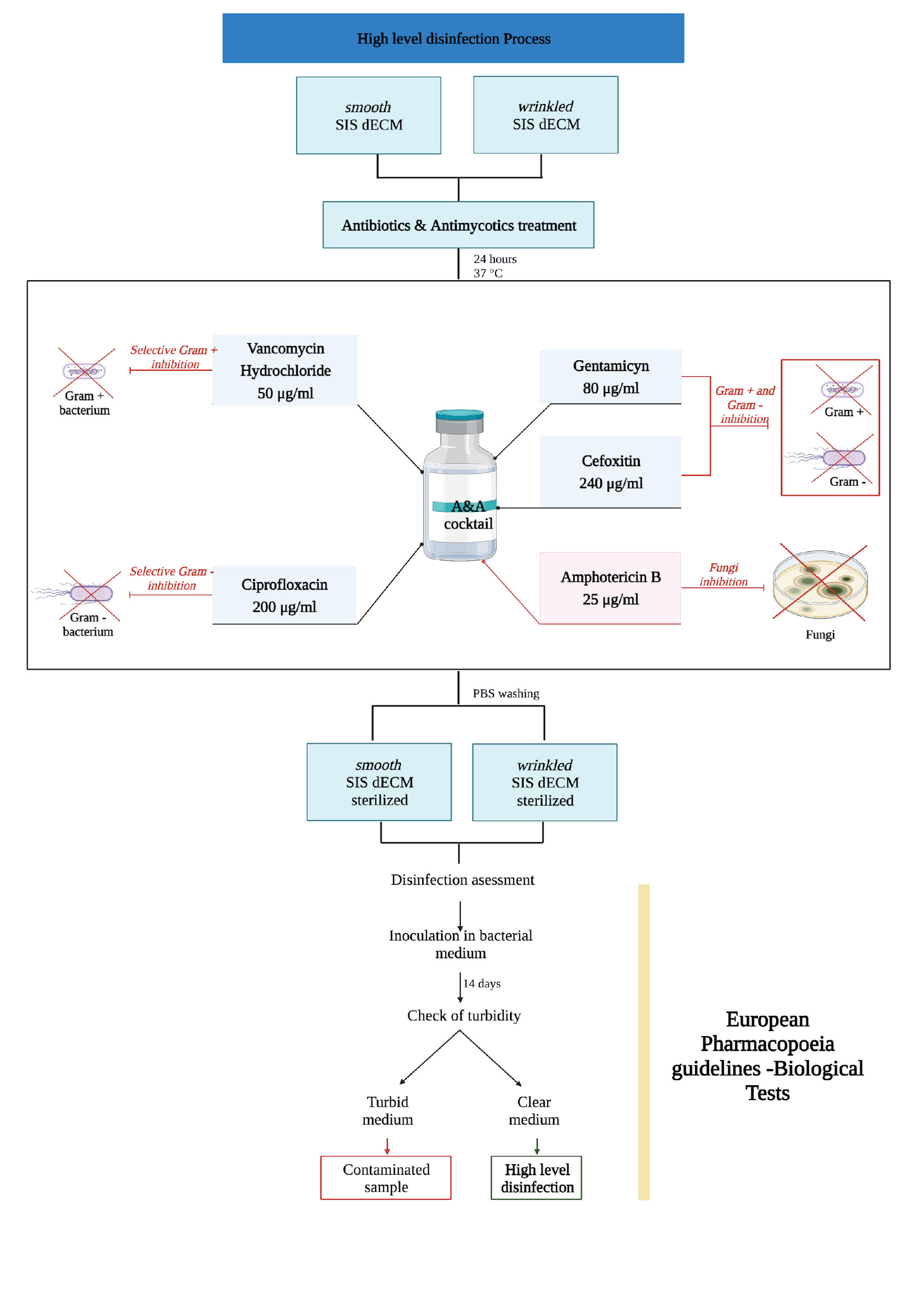
Figure S1 a: Flow chart of high-level disinfection process with Antibiotics & Antimycotics (A&A). Smooth and wrinkled SIS dECM are subjected to a 24-hour sterilization treatment at 37°C with antibiotics and antimycotics (A&A) (Fidalgo et al., 2018). Vancomycin Hydrochloride (50 μg/ml), Gentamycin (80 μg/ml), Cefoxitin (240 μg/ml) with the addition of Ciprofloxacin (200 μg/ml) for SIS tissue and Amphotericin B (25 μg/ml). PBS washes are necessary to remove excess drug from the tissue. Finally, sterility tests are performed following the European Pharmacopoeia. In this test, the tissue is inoculated into bacterial growth medium for 14 days and at the end, by visual inspection, the medium is assessed: if it is turbid, it is contaminated, if it is clear, it is a high-level disinfection sample.

Supplementary Figure 1

Supplementary Figure 2

2


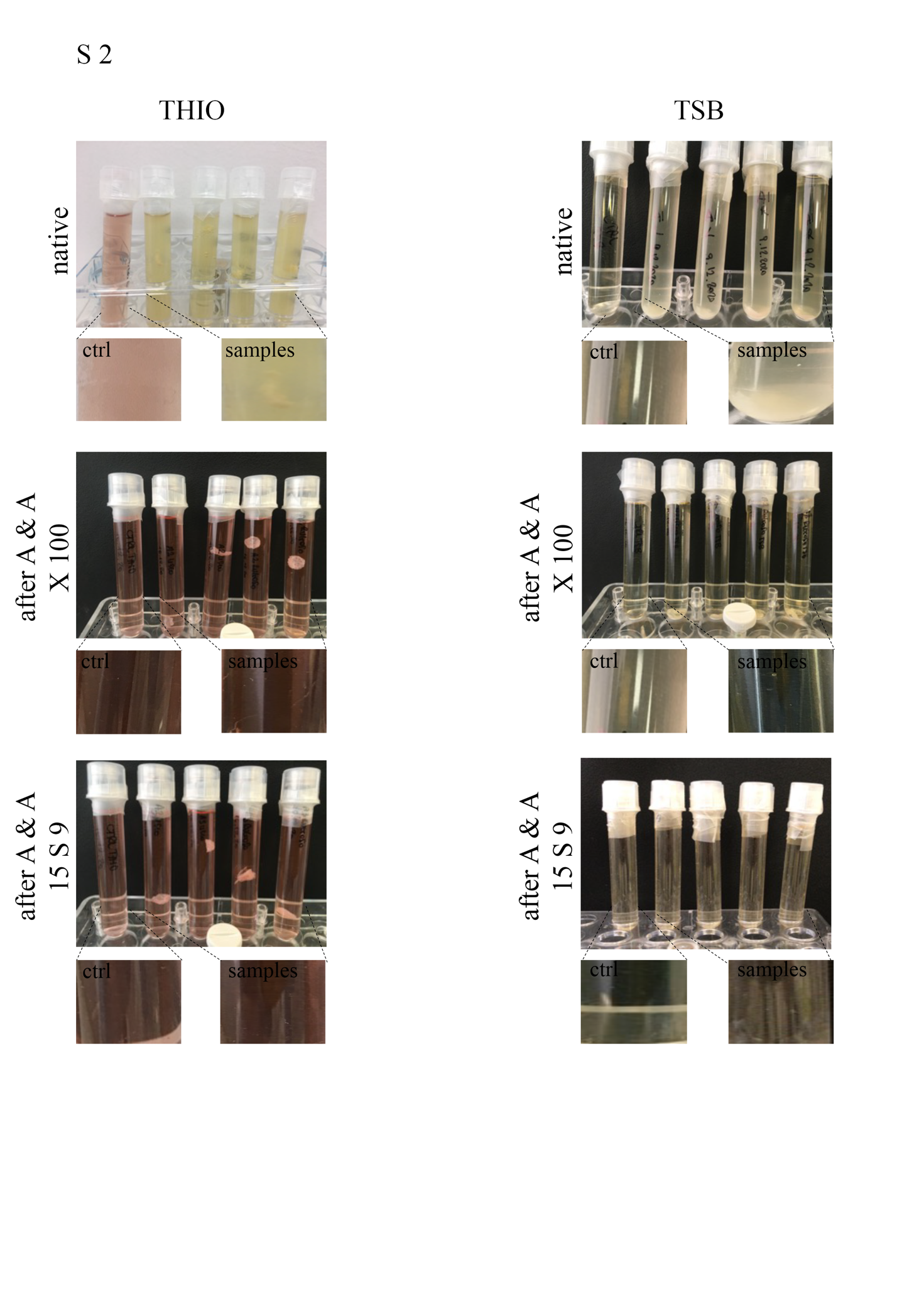


Figure S2 b: Decontamination test results. Tissues of smooth and wrinkled SIS, native and after the A&A process in X 100 and 15 S 9, (n=3 for each treatment) were inoculated for 14 days into the bacterial culture medium THIO (Thioglycolate, for the growth of anaerobic and aerobic microorganisms,) and TSB (Tryptic soy Broth, useful for the growth of fungi but also of aerobic and anaerobic bacteria). On visual analysis, THIO is red and when contaminated, the increase in oxygen concentration changes to yellow. TSB on the other hand is light yellow and clear, the increase in turbidity, as a result of uncontrolled osmotic balance, indicates growth of fungi and bacteria. After 14 days, bacterial contamination was present in the native: on visual inspection, THIO had turned yellow, from red, whereas after A&A and in the smooth and wrinkled X 100 and 15 S 9 scaffolds, it was still red, therefore sterile. Similarly, TSB in the native after 14 days was no longer clear but cloudy, contaminated. After A&A, in X 100 and 15 S 9, smooth and wrinkled, the TSB remained clear, sterile.

References:

European Pharmacopoeia 5.0, 2.6 Biological tests, 2.6.1 Sterility.01/2005:20601

Fidalgo, C., Iop, L., Sciro, M., Harder, M., Mavrilas, D., Korossis, S., et al. (2018). A sterilization method for decellularized xenogeneic cardiovascular scaffolds. *Acta Biomater* 67, 282–294. doi: 10.1016/j.actbio.2017.11.035.
